# Live reef fish displaying physiological evidence of cyanide poisoning are still traded in the EU marine aquarium industry

**DOI:** 10.1038/s41598-017-04940-x

**Published:** 2017-07-26

**Authors:** Marcela C. M. Vaz, Valdemar I. Esteves, Ricardo Calado

**Affiliations:** 1 Departamento de Biologia & CESAM, Universidade de Aveiro, Campus de Santiago, 3810-193 Aveiro, Portugal; 2 Departamento de Química & CESAM, Universidade de Aveiro, Campus de Santiago, 3810-193 Aveiro, Portugal

## Abstract

The illegal use of cyanide poisoning to supply live reef fish to several markets is one of the main threats to coral reefs conservation in the Indo-Pacific. The present study performed the first survey ever monitoring the marine aquarium trade in the EU for the presence of physiological evidence consistent with cyanide poisoning in live reef fish. This survey was also the first one worldwide employing a non-invasive sampling approach. Nearly 15% of the fish screened displayed physiological evidence of being illegally collected using cyanide poisoning (by testing positive for the presence of the thiocyanate anion (SCN^−^) in their urine). The efforts promoted so far to completely eradicate cyanide caught fish from the marine aquarium trade have not been effective, as our results suggest that their prevalence in the trade is in line with data reported nearly two decades ago. A new paradigm is urgently needed to effectively ban cyanide caught fish from the marine aquarium trade.

## Introduction

Coral reefs worldwide are endangered due to an unprecedented level of direct and indirect anthropogenic threats that may push these ecosystems beyond a point of no return^1^. Among the threats impacting coral reefs in the Indo-Pacific region, destructive fishing practices alone are considered to be the ones most severely affecting countries such as Indonesia and the Philippines^2^. These two countries are known to be among the main suppliers of live reef organisms entering the marine aquarium trade, a multi-million dollars industry acting at a global scale and having the USA and the EU as their main importing markets^3, 4^. Cyanide fishing, one of the most destructive fishing techniques employed in Indo-Pacific coral reefs to collect live fish, is commonly, but not exclusively, employed to supply the marine aquarium trade, with its use in Indonesia and the Philippines being well documented^5^. For a detailed description of this fishing technique please see Rubec *et al*.^6^. Cyanide fishing has been officially banned several years ago in most Indo-Pacific countries, including Indonesia and the Philippines^5^. Nonetheless, these regulations are still poorly enforced by exporting and importing countries^7^, with this destructive fishing practice continuing to be employed to supply an undetermined number of live reef fish to importing markets.

The majority of wholesalers trading live reef fish for marine aquariums claim to support responsible collection practices and often exhibit pseudo-certification stating that no specimens collected using cyanide fishing are traded by their companies^8^. However, the challenges associated with the traceability of traded marine ornamental fish are well-documented^9^, being very difficult to reliably trace the origin of a given specimen along the blurry supply chains that characterize this industry. The lack of a suitable methodology that may allow the screening of live reef fish collected with cyanide without requiring the sacrifice of these highly priced animals^10^ has also contributed for the *status quo* in the marine aquarium trade – a general perception by traders that cyanide fishing no longer plays a role on the supply of the global trade of marine ornamental fish and a certain over dimensioning of this issue by marine conservationists in their quest to ban the collection of live fish from coral reefs. The non-invasive and non-destructive screening approach proposed by Vaz, *et al*.^11^ to detect live reef fish collected with cyanide fishing has started to shift the perception of the whole chain of custody on the true dimension that this destructive fishing practice may have in the marine aquarium trade. This approach screens for the presence of the thiocyanate anion (SCN^−^) in the urine of fish, a metabolite originating from the main metabolic pathway involved in the detoxification process displayed by vertebrates poisoned by the cyanide anion (CN^−^)^12^. Fish being screened must be stocked in clean synthetic seawater (with no detectable levels of SCN^−^) where the specimens being surveyed are depurated for 24 h (or in other words allowed to urinate); following this period a sample of that water (only a few mL) is screened for the presence of SCN^−^ (in the range of ug L^−1^), whose source can only be the fish being depurated^11^. This non-destructive approach is likely to be more readily accepted by the industry than alternative techniques already available to detect live reef fish poisoned by cyanide, which use as a screening matrix fish blood or muscle and target the presence of the less persistent anion CN^−^^13^.

The objective of the present work was to perform the first survey on physiological evidence of cyanide poisoning in marine ornamental fish being imported from the Indo-Pacific region through well-established commercial channels supplying the EU marine aquarium trade. It is important to highlight that this is the first study ever performed to screen live reef fish for potential cyanide poisoning where no animals being traded needed to be sacrificed, unlike previous screening programs implemented in the past (see ref. 14).

## Results

**Figure 1:**
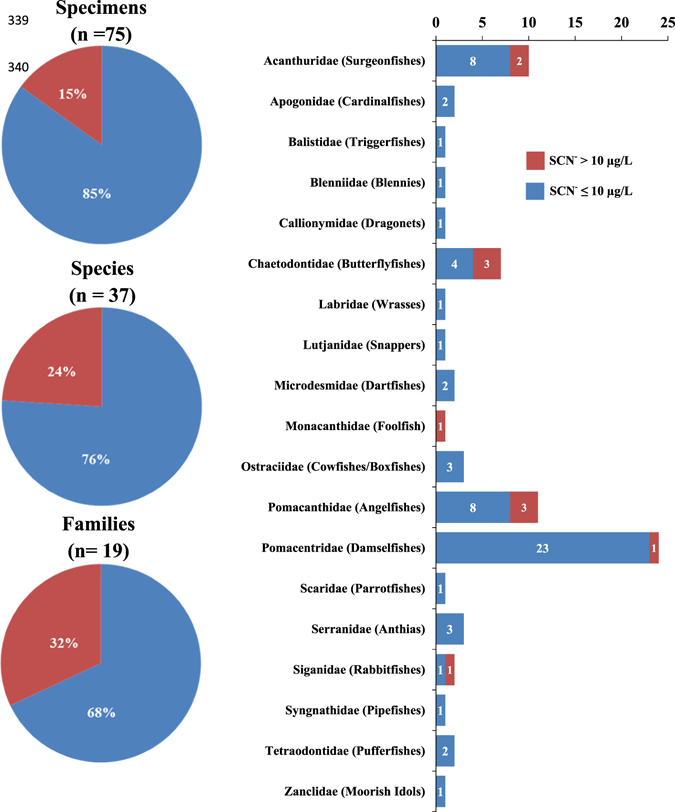
Percentage of reef fish specimens, species, and families (pie charts) and number of specimens per fish family (bar chart) displaying concentrations of SCN^−^ >10 µg/L in the water at the end of the depuration period (24 h).

A total of 75 fish specimens representing 37 different species from 19 families were screened in the present study (see Supplementary Table S1). From all screened fish, a total of 11 specimens, representing 9 different species from 6 different families, were recorded excreting SCN^−^ at concentrations >10 µg L^−1^ at the end of the depuration procedure (Fig. 1; see Supplementary Table S1 and Supplementary Figure S1 for additional information). In other words, 15% of all screened fish, representing nearly 1/4 of all species and 1/3 of all families screened were flagged as showing physiological evidence of having been poisoned by cyanide. Families Chaetodontidae (butterflyfishes), Pomacanthidae (angelfishes) and Acanthuridae (surgeonfishes) were the ones displaying the highest number of specimens (3, 3 and 2, respectively) excreting SCN^−^ at concentrations >10 µg L^−1^ at the end of the screening.

## Discussion

The present study revealed that reef fish displaying physiological evidence of having been illegally collected using cyanide fishing are still present in the marine aquarium trade in the EU. Even when employing a very conservative approach, by only considering a fish as testing positive for potential cyanide poisoning when the recorded concentration of SCN^−^ was >10 µg L^−1^, our study revealed that 15% of all screened specimens displayed physiological evidence that they had been exposed to this toxic. Nonetheless, it is worth highlighting that physiological evidence of cyanide poisoning is not an absolute demonstration of illegal fishing, as a fish may survive an acute episode of exposure to cyanide and a few days latter be captured in the reef by a licensed collector using a hand net - this fish would be a case of a legally harvested organism that would display physiological evidences of cyanide poisoning.

The results reported in the present work are in line with those reported nearly two decades ago by Rubec, *et al*.^14^ for marine aquarium fish surveyed in the Philippines between 1996 and 2000. These authors, which have employed a different methodological approach, report a proportion of fish testing positive for cyanide poisoning ranging from 8 to 43% during the period surveyed. The three fish families recorded in our study with the highest number of contaminated specimens (Chaetodontidae, Pomacanthidae and Acanthuridae), were also ranked among the top 10 families displaying the highest percentage of fish testing positive for CN^−^ in the study by Rubec, *et al*.^14^. The efforts promoted by governmental agencies and the marine aquarium trade to eradicate cyanide caught fish from the selling lists of suppliers have not been fully effective over all these years. Some of those efforts have claimed the implementation of responsible collection practices and even, at times, offered a pseudo-certification of the players involved in the supply chain^8^.

The date of collection and the accurate dosage of cyanide used to collect a fish being surveyed are impossible to determine given the lack of reliable traceability protocols in this industry^9^. The cyanide solution in the squirt bottles of fishermen employing this technique becomes diluted as the mix is squirted and seawater enters the squirt bottle. Initial concentrations can be as high as 1400 mg L^−1^ of CN^−^^15^, thus a fish collected in the beginning of the fishing journey is exposed to a higher dosage of CN^−^ and likely starts excreting SCN^−^ sooner and in higher levels than an identical specimen poisoned later during the fishing journey. The latter a fish is screened for SCN^−^, in terms of post collection days, the lower the chances of physiological evidences for cyanide poisoning being recorded using the methodology reported. Other issues that are still poorly understood and may condition the time frame available for the detection of cyanide poisoning are related with the role that fish biomass and species may play on the efficiency of the metabolic pathway involving the enzyme rhodanese (thiosulfate sulfurtransferase), which in the presence of a sulfur donor converts CN^−^ to SCN^−^. As discussed by Vaz, *et al*.^11^, by increasing the holding period of collected fish in their facilities, traders may eventually avoid detection using the approach employed in our study, as specimens can be shipped when no longer excreting SCN^−^ (when fully detoxified). However, traders doing so also risk losing these fish due to the higher mortality commonly associated with specimens collected with cyanide^6^. To date, there is no scientific data available to either confirm or refute the potential cross contamination of non-cyanide caught reef fish stocked in the same tank with fish illegally collected with cyanide and actively excreting SCN^−^. However, if one considers that fish exposed to mg of CN^−^ excrete µg of SCN^−^ (see Vaz, *et al*.^11^), it is unlikely that a fish in contact with other specimens actively excreting µg of SCN^−^ will be cross-contaminated to the point of also excreting SCN^−^ within the same concentration range. In the present study, this potential issue was controlled by only considering as positive the water samples collected from specimens excreting 10 µg L^−1^ of SCN^−^. Overall, by monitoring the excretion of SCN^−^ in live reef fish being traded to supply marine aquariums, and also taking into account the numbers of fish being rejected along the supply chain^16^, the number of specimens potentially being signaled as originating from cyanide fishing will likely underestimate the impact of this illegal practice in this industry.

The methodological approach employed in the present work may not be the most suitable one to be scaled up by the industry due to the need to employ synthetic seawater, depurate each target fish individually and use an HPLC approach that requires highly trained staff (e.g., operator must know how to interpret shifts on SCN^−^ retention times and determine when the stripping of Polyethyleneglycol (PEG) used to modify the C30 column has reached a point at which the column needs to be once again modified with this compound to allow the reliable detection of SCN^−^). Indeed, the method employed in our study may likely only be suitable for academic purposes to better understand the dynamics of SCN^−^ excretion over time in different live reef fish exposed to different concentrations of CN^−^. This method may also be employed by a specialized sub-contracted laboratory specifically to screen a restricted number of fish (a few tens) originating from an import over which there are strong suspicions of trading specimens illegally collected with cyanide. Overall, we advocate that the focus of future studies should be on alternative non-invasive and non-destructive methodologies which allow an easier and faster detection of the active excretion of SCN^−^ or other relevant metabolites in natural seawater by fish poisoned by cyanide.

## Methods

Over 600 marine ornamental fish were purchased from three different major EU wholesalers from May 2014 to June 2015 importing fish from the main exporting nations in the Indo-Pacific region supplying this industry (see ref. 4) and distributing them to all EU member states, as well as other non-member states (e.g., Switzerland, Norway and Russia). None of the suppliers was previously informed about the survey being performed, in order to avoid any shift in their *modus operandi*. All specimens received at the University of Aveiro (Portugal) were individually packed in plastic bags sealed with rubber bands (or metal clips), with 1/3 of the bag volume being filed with seawater and the other 2/3 being an oxygen saturated atmosphere. Immediately upon arrival all fish were acclimatized using the drip method (see Calado^17^ for a detailed description) and fed following standard procedures. Two days after acclimatization, fish species known to occur in the Indo-Pacific region (where cyanide fishing is employed to collect marine ornamental fish) were haphazardly selected to be screened for SCN^−^ excretion. It is important to highlight that the lack of a reliable traceability protocol to survey the supply chain of marine ornamental fish^9^ does not allow to pinpoint the place of collection (or at times even the country of origin). Selected fish were submitted to a 24-h depuration process, following the methodology described by Vaz *et al*.^11^. Briefly the depuration process can be described as follows: fish haphazardly selected for screening were housed in individual glass jars filled with clean synthetic seawater prepared by mixing freshwater purified by reverse osmosis with a synthetic salt mix (Tropic Marine^®^ Pro Reef salt); the volume of seawater employed to depurate each fish was adjusted according to the fish total length (measured from the tip of the snout to the tip of the longer lobe of the caudal fin) (see Supplementary Table S2). During the depuration process fish were not fed and were exposed to a 12 h L: 12 h D photoperiod. The use of synthetic seawater was preferred over the use of natural seawater to prevent the presence of SCN^−^ in the depuration water (thus minimizing the risks of detecting false positives) and reduce the potential interference of other compounds with SCN^−^ when performing the HPLC (High-Performance Liquid Chromatography) analysis (which could impair the detection of SCN^−^ and give origin to false negatives). All batches of synthetic seawater prepared for the present study were screened for the presence of SCN^−^ (see below for methodological details on this analysis) in order to ensure that they were free of any detectable levels of this compound and that no false positives were detected. Water quality parameters at the end of the depuration procedure were always recorded at the following values: pH 8.1 ± 0.1; undetectable levels of ammonium and nitrite, with nitrate always being recorded <5 mg L^−1^. Water temperature was stable at 26 ± 0.5 °C and the salinity recorded was 35 ± 1. A sample of 5 mL of the seawater used to depurate each fish was sampled from each individual jar before receiving the fish, as well as 24 h after the depuration procedure; seawater samples were stocked in plastic Eppendorf tubes and immediately stored at −20 °C until they were used for screening the presence of SCN^−^ using HPLC.

Prior to HPLC analysis, samples were defrosted, submitted to an ultrasonic bath for 60 minutes and filtered through a PTFE (Polytetrafluoroethylene) syringe filter with a pore diameter of 0.22 µm. Once this procedure was complete 1.5 ml of each seawater sample was transferred to an individually labeled HPLC glass vial to proceed with the analysis. The analysis were performed using a HPLC (Waters 2695) equipped with a C30 packed column (100 mm × 0.32 mm i.d.) modified with PEG (Polyethyleneglycol − H(OCH_2_CH_2_)_n_OH − Brand Alfa Aeasar) 5%, following the methodology described by Rong *et al*.^18^. However, in the present study, 350 mM Sodium sulfate was used as mobile phase. A UV-VIS (Ultraviolet-visible spectrophotometry) detector (Waters 2487 Dual λ Absorbance Detector) was used at 220 nM and retention times recorded for the SCN^−^ were always between 4.2 to 4.8 minutes. A minimum of 3 injections per sample was performed, with calibration deviation always being <5%. The detection limit was between 2.56 and 3.44 µg L^−1^ SCN^−^ and only for results >10 µg L^−1^ SCN^−^ was a fish considered as testing positive for cyanide exposure. This very conservative approach was employed to avoid flagging false positives. During the HPLC analysis all samples were kept in the dark and at room temperature. Samples of the synthetic seawater prepared by mixing freshwater purified by reverse osmosis with a synthetic salt mix (Tropic Marine^®^ Pro Reef salt) used to depurate the fish were also used as a negative control (no detectable concentrations of SCN^−^). A stock solution of CNNaS (Fluka, 98% purity), with a SCN^−^ concentration of 40 mg L^−1^, was prepared by dissolving 5.697 mg L^−1^ of CNNaS in 100 mL of Milli-Q water, which was used to prepare five standard solutions with concentrations of 5, 10, 20, 40 and 60 µg L^−1^ of SCN^−^ in synthetic seawater (see above for details) and used for HPLC calibration. All standard solutions of SCN^−^ were submitted to exactly the same procedures described above for seawater samples prior to analysis in the HPLC.

Whenever a sample of seawater collected after depuration revealed the presence of SCN^−^ the following additional analysis were performed: the sample being analyzed, as well as a sample from the seawater prepared for depurating the fish, was spiked using standard solutions of 10 and 40 µg L^−1^ of SCN^−^ and analyzed in the HPLC. This procedure was used as a quality check for accuracy and to avoid signaling false positives: (1) it ensured that the SCN^−^ concentration initially reported from the first HPLC analysis of the seawater sample screened after depuration was indeed accurate (SCN^−^ concentration would have to be X + 10 and X + 40 µg L^−1^ of SCN^−^, with X being the concentration of SCN^−^ being reported in the first HPLC analysis); and (2) that the sample was not a false positive and that the SCN^−^ being recorded was indeed coming from fish excretion and not from the seawater prepared to depurate the fish (SCN^−^ concentration on the water prepared for depurating the fish would have to be 10 and 40 µg L^−1^ of SCN^−^, the concentrations of the standard solutions used for spiking the sample) (see Supplementary Figure S2).

Scientific names of fish species and families are reported according to WoRMS^19^, while common names are reported according to Michael^20^.

This study followed an experimental protocol approved by the Commission Responsible for Animal Experimentation and Welfare (CREBEA) of the Department of Biology of the University of Aveiro and performed in strict accordance with the Guidelines of the European Union Council (Directive 2010/63/EU) and the Portuguese legislation for the use of laboratory animals; the study was conducted under an institutional license for animal experimentation and a personal license to M.C.M. Vaz issued by the Veterinary Medicine Directorate, Portuguese Ministry of Agriculture, Rural Development and Fisheries.

## Electronic supplementary material

Supplementary Information
