# Supplementary material for: Live reef fish displaying physiological evidence of cyanide poisoning are still traded in the EU marine aquarium industry
Source: Sci Rep. 2017 Jul 26;7:6566. doi: 10.1038/s41598-017-04940-x (PMC5529361; doi:10.1038/s41598-017-04940-x)
Supplement: Supplementary file 1 — Supplementary Information [file 41598_2017_4940_MOESM1_ESM.pdf]

Live reef fish displaying physiological evidence of cyanide poisoning are still traded in the EU marine aquarium industry

Marcela C.M. Vaz[1,2], Valdemar I. Esteves[2], Ricardo Calado[1]*

[1] Departamento de Biologia & CESAM, Universidade de Aveiro, Campus de Santiago, 3810-193 Aveiro, Portugal.

[2] Departamento de Química & CESAM, Universidade de Aveiro, Campus de Santiago, 3810-193 Aveiro, Portugal.

*Corresponding author: Ricardo Calado

Phone: +351 234370779

E-mail: rjcalado@hotmail.com

Supplementary Figure S1 - Chromatograms performed using HPLC to screen for the presence of SCN^- in seawater used to depurate live reef fish traded for marine aquariums. All details concerning each chromatogram are summarized in Supplementary Table S1.

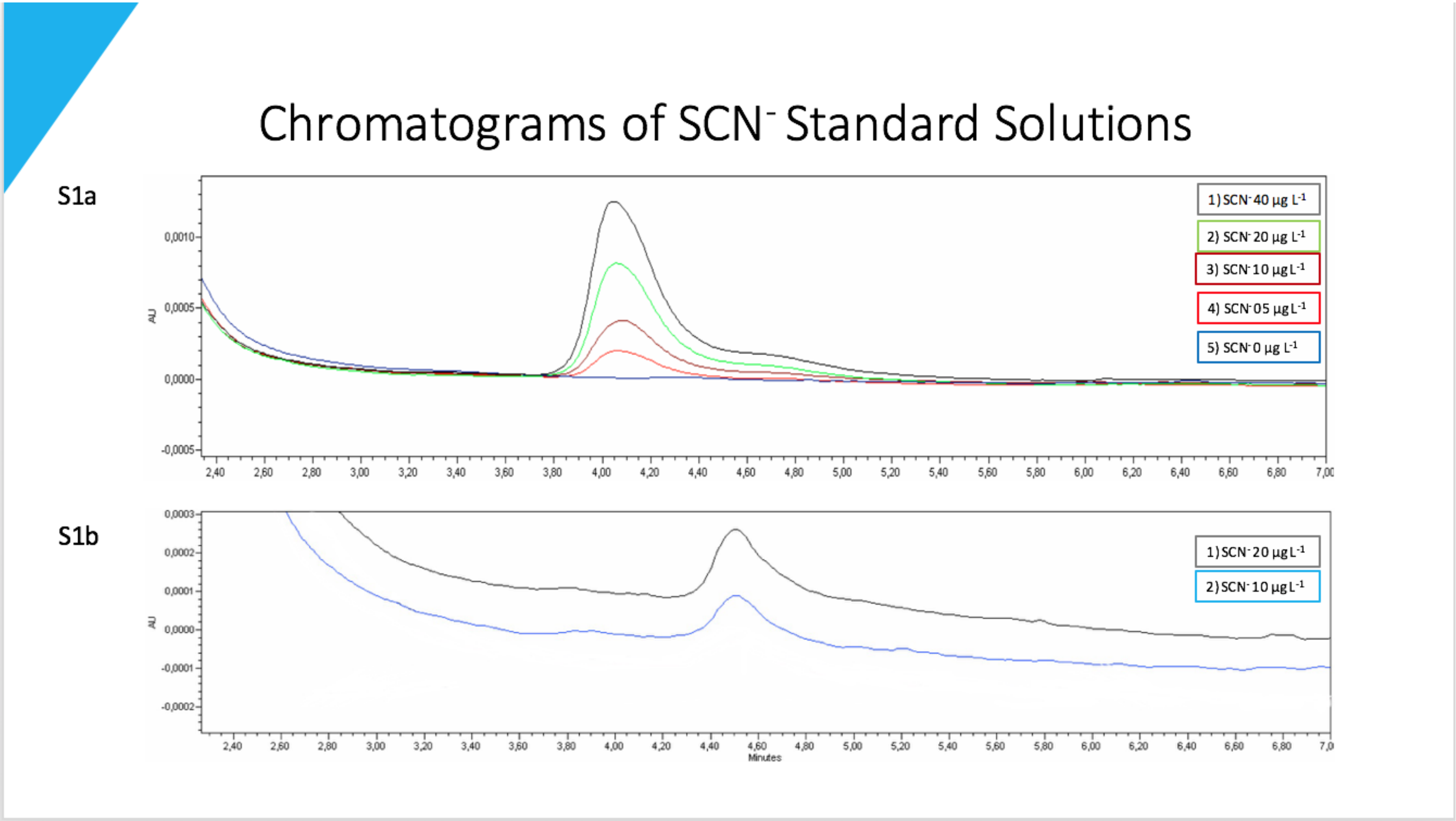

Chromatograms tested positive for SCN^-

S1c

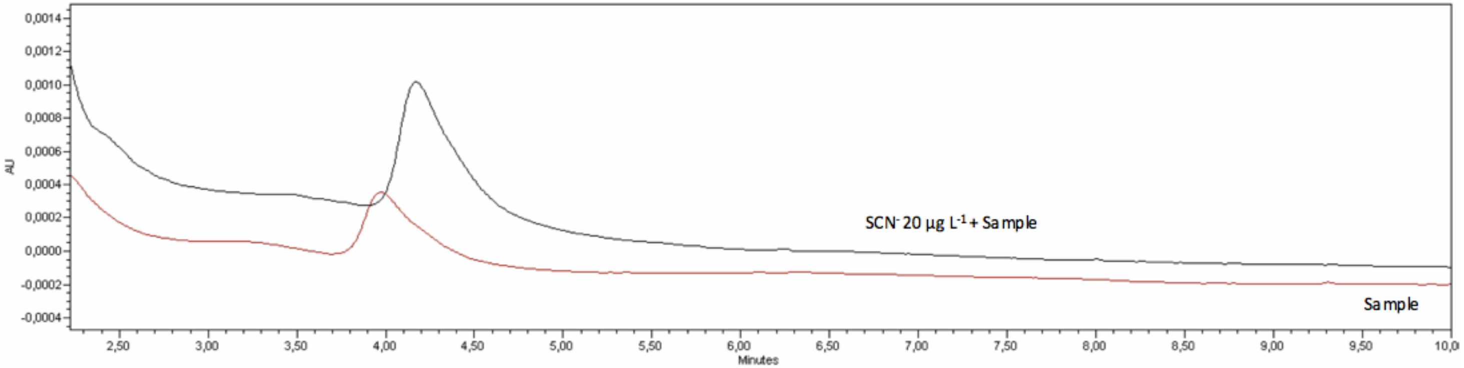

S1d

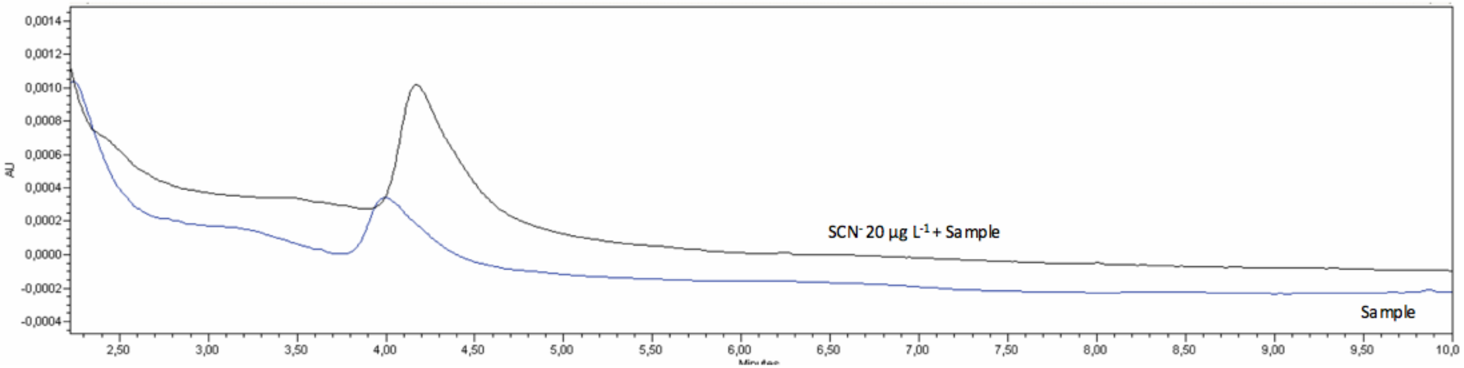

S1e

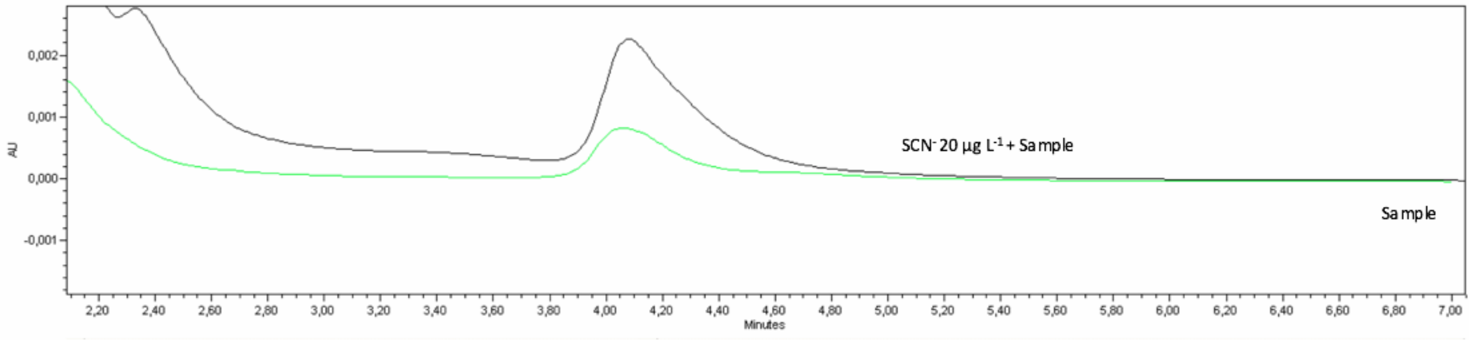
0,002
0,001
0,000
-0,001
AU
2,20 2,40 2,60 2,80 3,00 3,20 3,40 3,60 3,80 4,00 4,20 4,40 4,60 4,80 5,00 5,20 5,40 5,60 5,80 6,00 6,20 6,40 6,60 6,80 7,00
Minutes
SCN- 20 µg L-1 + Sample
Sample

S1f

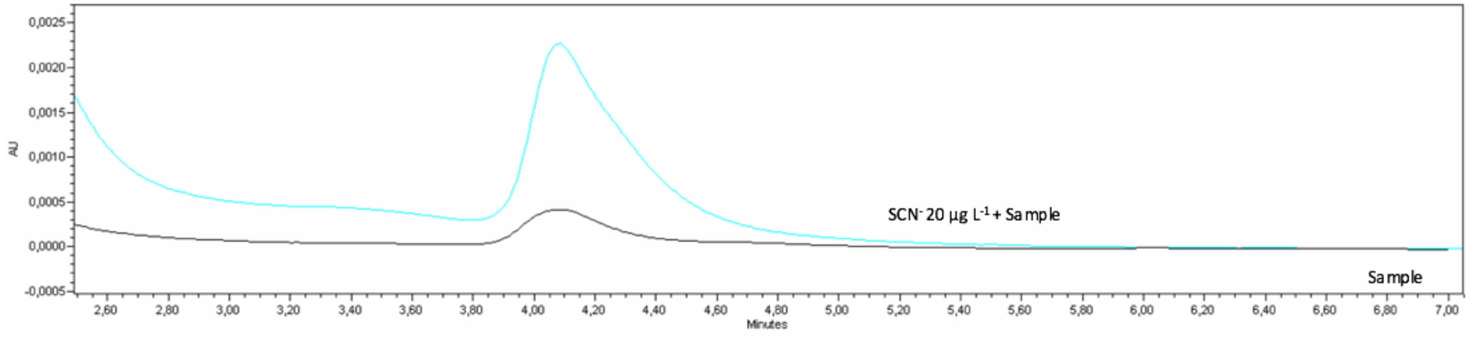
0,0025
0,0020
0,0015
0,0010
0,0005
0,0000
-0,0005
AU
2,60 2,80 3,00 3,20 3,40 3,60 3,80 4,00 4,20 4,40 4,60 4,80 5,00 5,20 5,40 5,60 5,80 6,00 6,20 6,40 6,60 6,80 7,00
Minutes
SCN- 20 µg L-1 + Sample
Sample

S1g

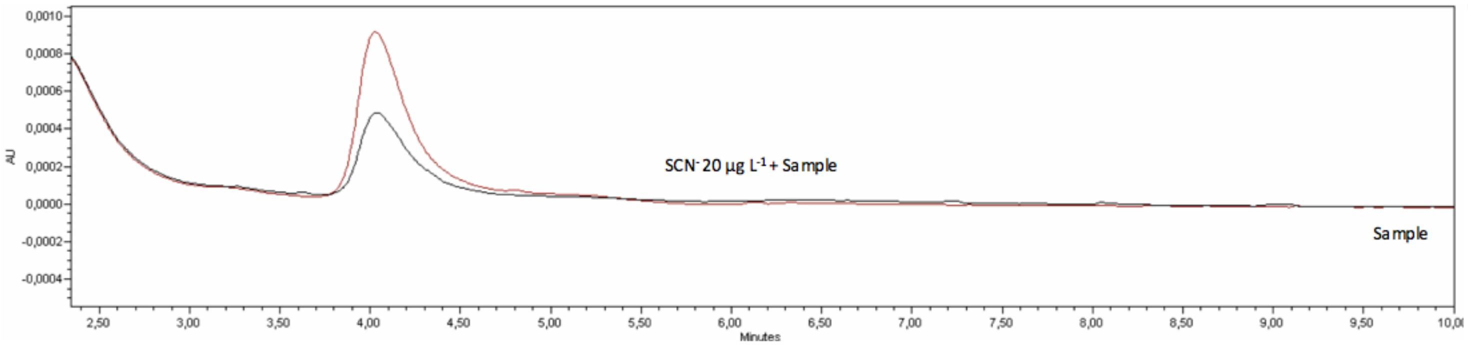
AU
Minutes
SCN- 20 µg L-1 + Sample
Sample

S1h

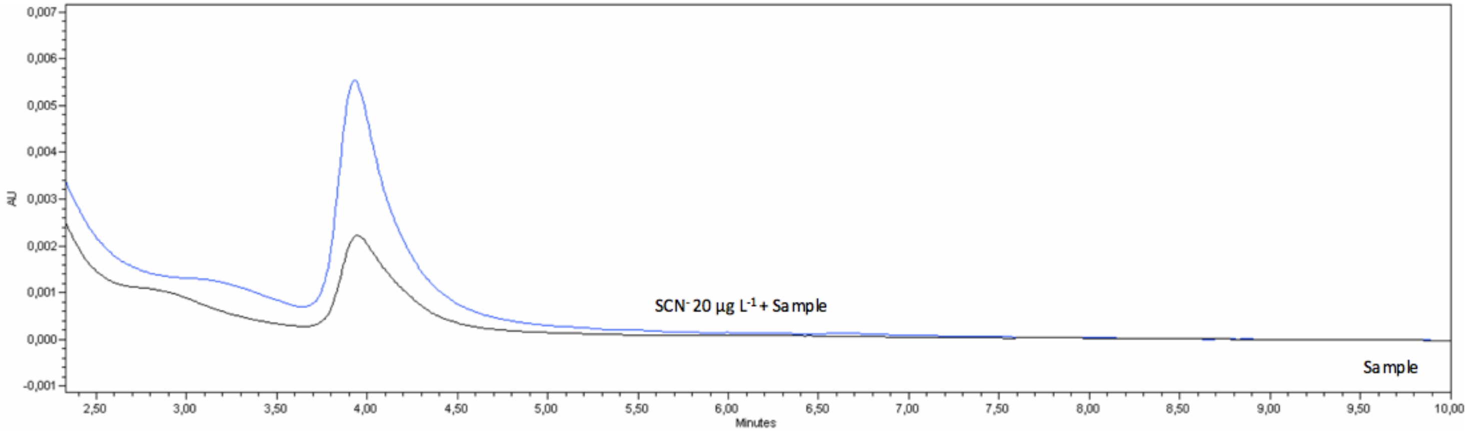
AU
Minutes
SCN- 20 µg L-1 + Sample
Sample

S1i

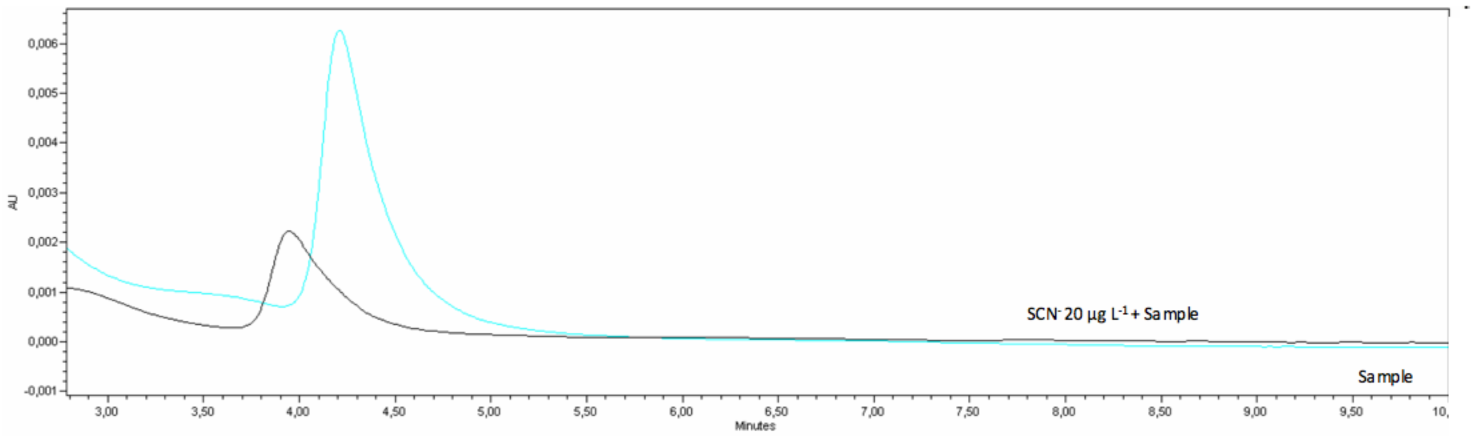
0,006
0,005
0,004
0,003
0,002
0,001
0,000
-0,001
AU
3,00
3,50
4,00
4,50
5,00
5,50
6,00
6,50
7,00
7,50
8,00
8,50
9,00
9,50
10,
Minutes
SCN- 20 µg L-1 + Sample
Sample

S1j

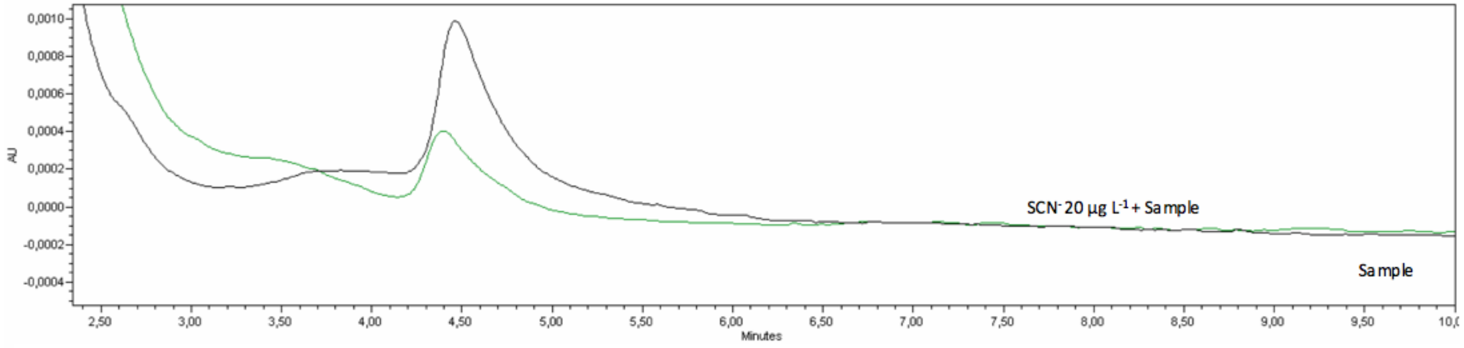
0,0010
0,0008
0,0006
0,0004
0,0002
0,0000
-0,0002
-0,0004
AU
2,50
3,00
3,50
4,00
4,50
5,00
5,50
6,00
6,50
7,00
7,50
8,00
8,50
9,00
9,50
10,(
Minutes
SCN- 20 µg L-1 + Sample
Sample

S1k

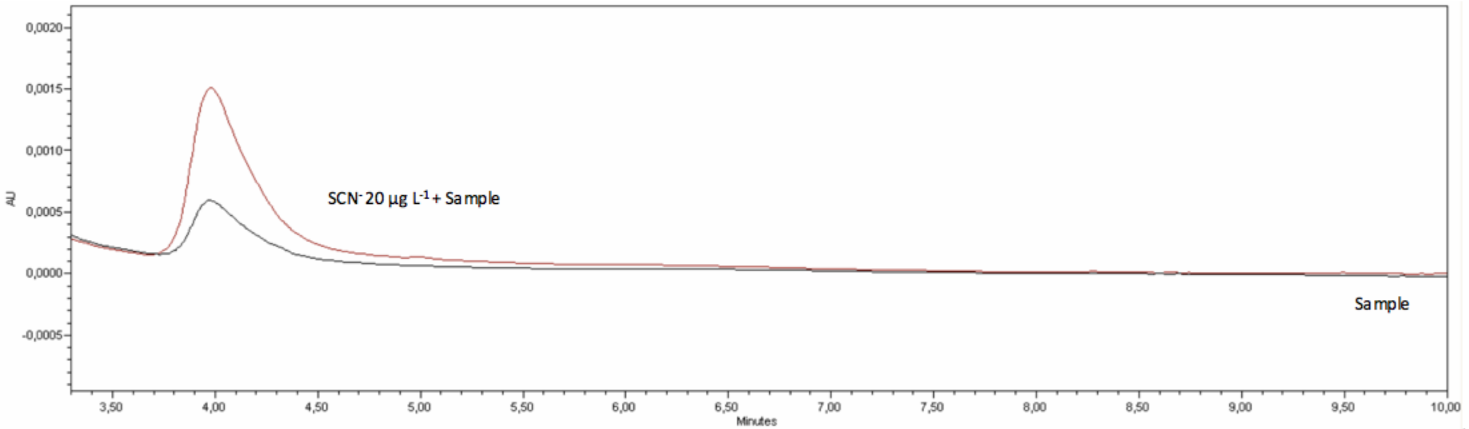

0,0020
0,0015
0,0010
0,0005
0,0000
-0,0005
AU
3,50 4,00 4,50 5,00 5,50 6,00 6,50 7,00 7,50 8,00 8,50 9,00 9,50 10,00
Minutes
SCN- 20 µg L-1 + Sample
Sample

S1l

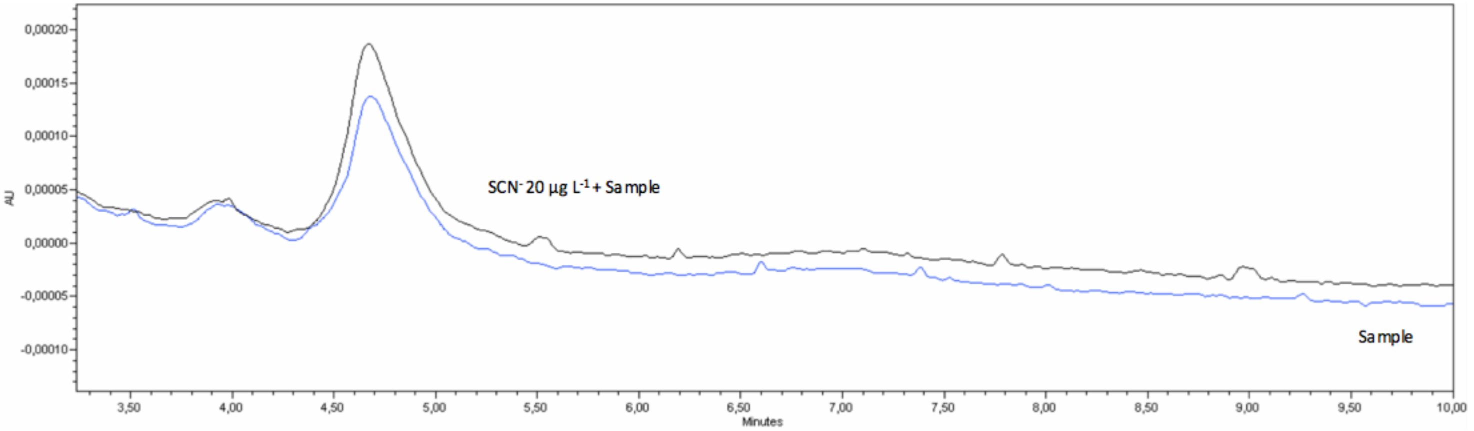

0,00020
0,00015
0,00010
0,00005
0,00000
-0,00005
-0,00010
AU
3,50 4,00 4,50 5,00 5,50 6,00 6,50 7,00 7,50 8,00 8,50 9,00 9,50 10,00
Minutes
SCN- 20 µg L-1 + Sample
Sample

Chromatograms tested negative for SCN^- * For concentrations above SCN^- 10 µg L^{-1}

S1m

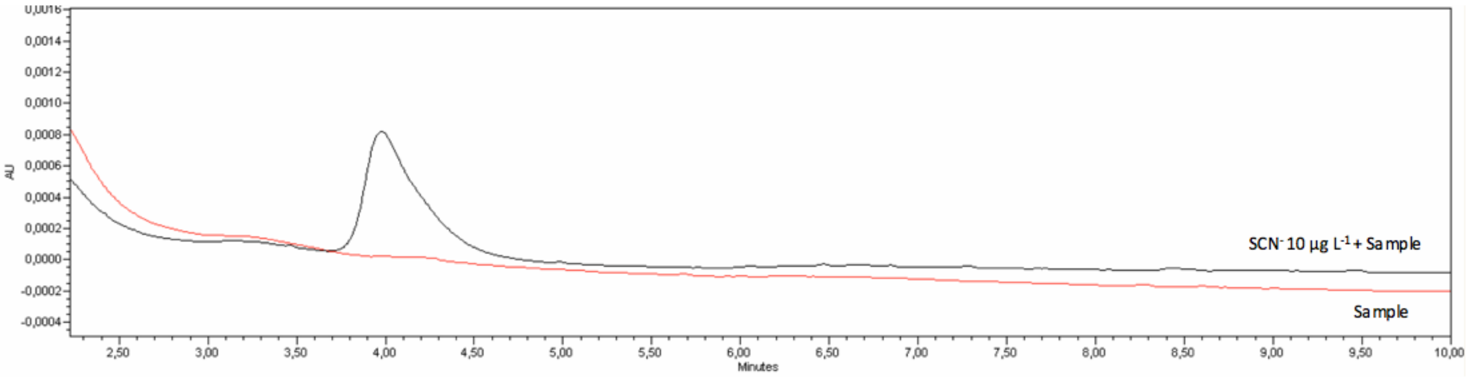

S1n

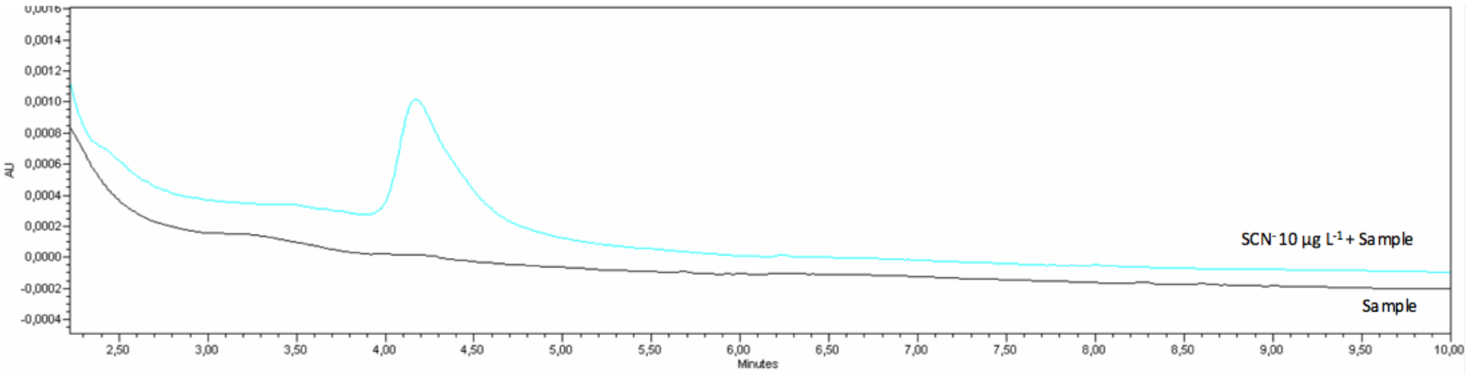

S1o

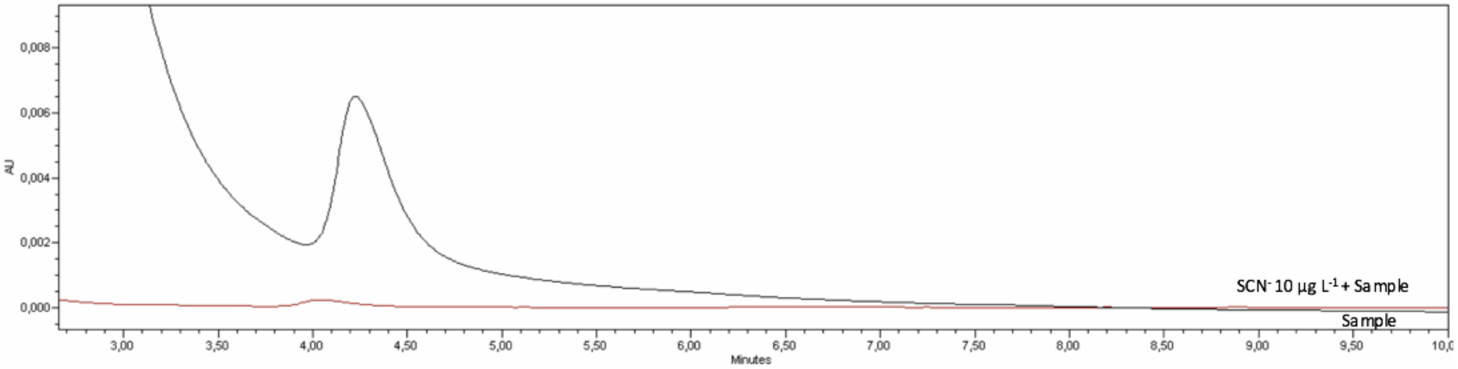
AU
0,000
0,002
0,004
0,006
0,008
Minutes
3,00
3,50
4,00
4,50
5,00
5,50
6,00
6,50
7,00
7,50
8,00
8,50
9,00
9,50
SCN- 10 µg L-1 + Sample
Sample

S1p

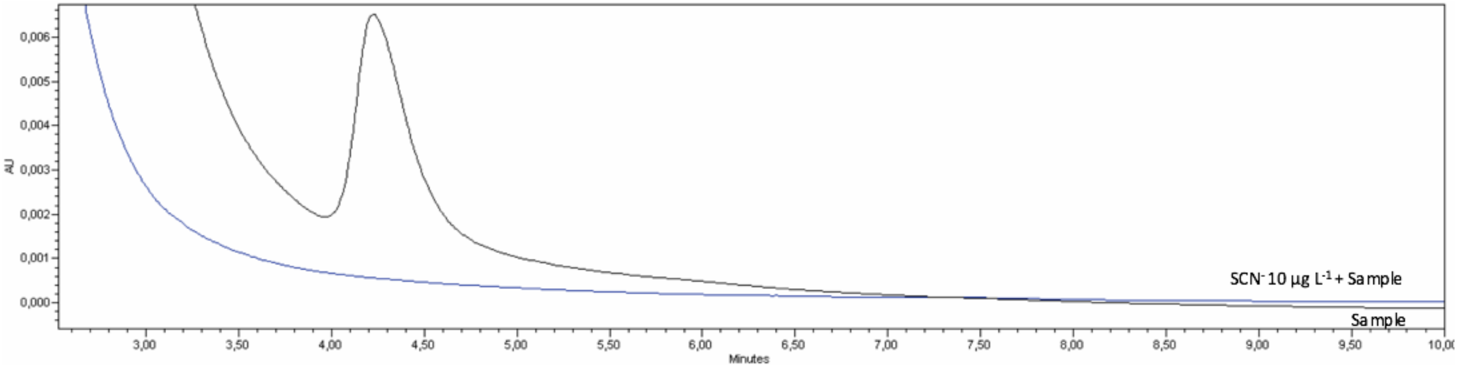
AU
0,000
0,001
0,002
0,003
0,004
0,005
0,006
Minutes
3,00
3,50
4,00
4,50
5,00
5,50
6,00
6,50
7,00
7,50
8,00
8,50
9,00
9,50
SCN- 10 µg L-1 + Sample
Sample

S1q

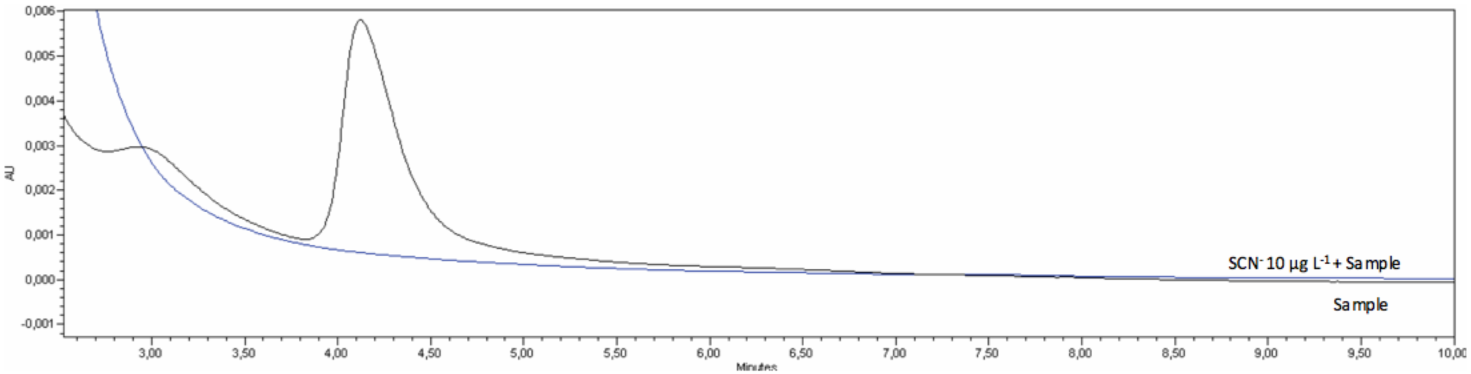
0,006
0,005
0,004
0,003
0,002
0,001
0,000
-0,001
AU
3,00
3,50
4,00
4,50
5,00
5,50
6,00
6,50
7,00
7,50
8,00
8,50
9,00
9,50
SCN- 10 µg L-1 + Sample
Sample

S1r

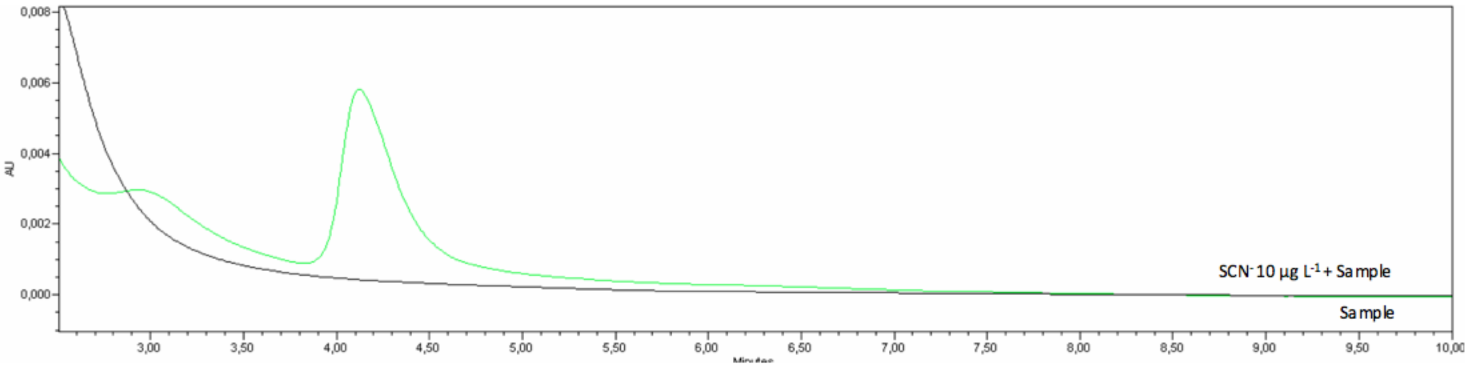
0,008
0,006
0,004
0,002
0,000
AU
3,00
3,50
4,00
4,50
5,00
5,50
6,00
6,50
7,00
7,50
8,00
8,50
9,00
9,50
SCN- 10 µg L-1 + Sample
Sample

S1s

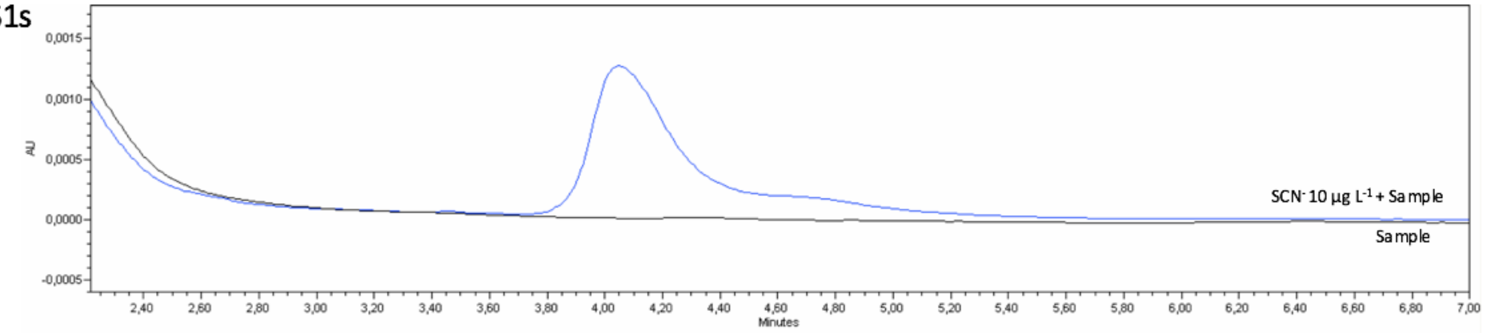
AU
0,0015
0,0010
0,0005
0,0000
-0,0005
2,40
2,60
2,80
3,00
3,20
3,40
3,60
3,80
4,00
4,20
4,40
4,60
4,80
5,00
5,20
5,40
5,60
5,80
6,00
6,20
6,40
6,60
6,80
7,00
Minutes
SCN- 10 µg L-1 + Sample
Sample

S1t

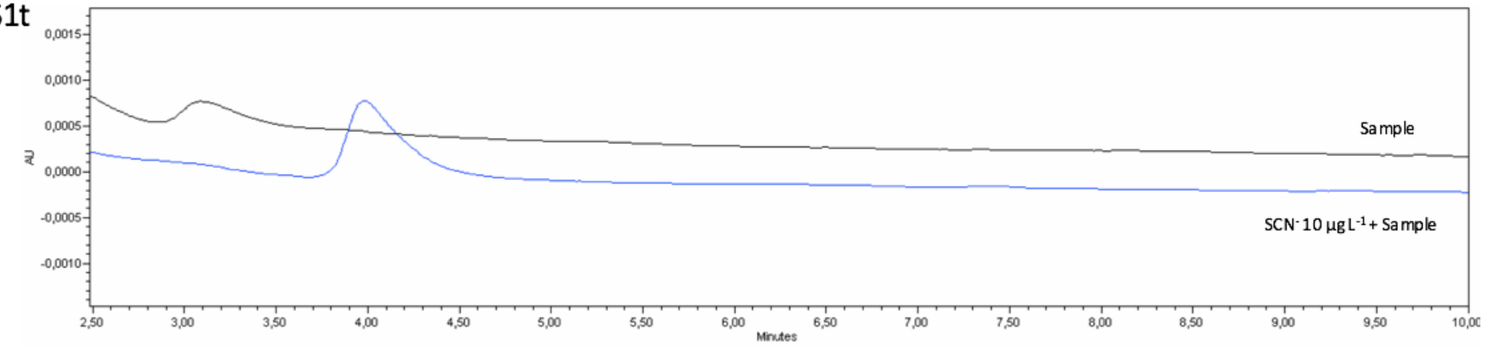
AU
0,0015
0,0010
0,0005
0,0000
-0,0005
-0,0010
2,50
3,00
3,50
4,00
4,50
5,00
5,50
6,00
6,50
7,00
7,50
8,00
8,50
9,00
9,50
10,00
Minutes
Sample
SCN- 10 µg L-1 + Sample

S1u

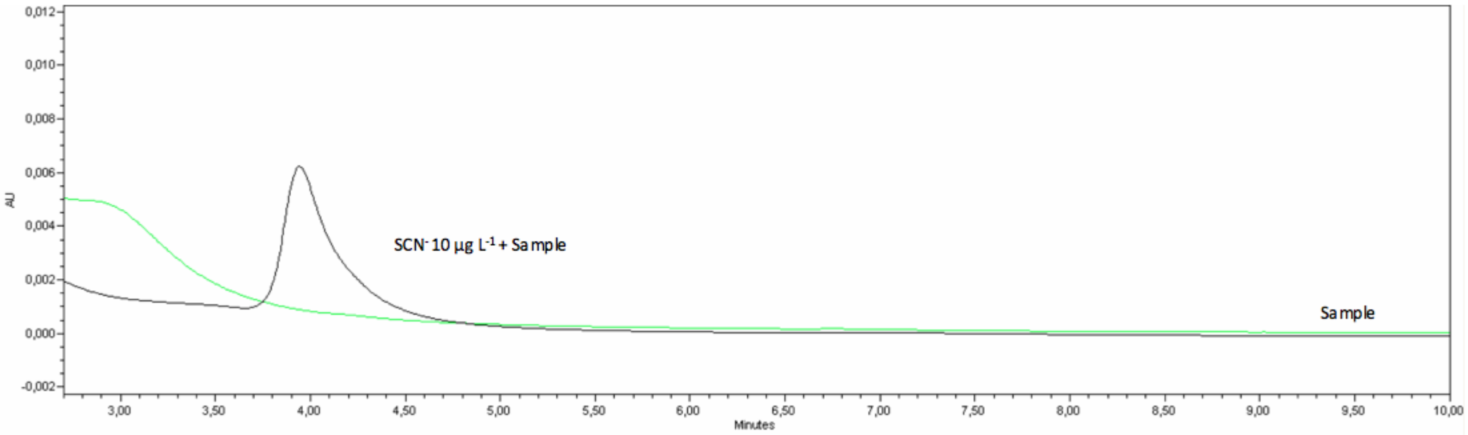

AU
Minutes
SCN- 10 µg L-1 + Sample
Sample

S1v

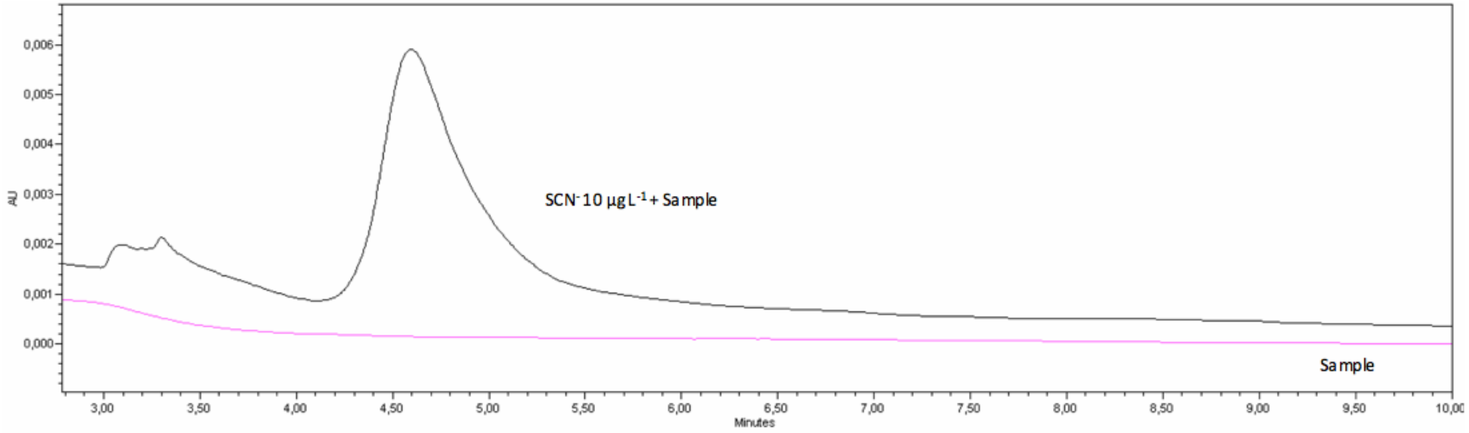

AU
Minutes
SCN- 10 µg L-1 + Sample
Sample

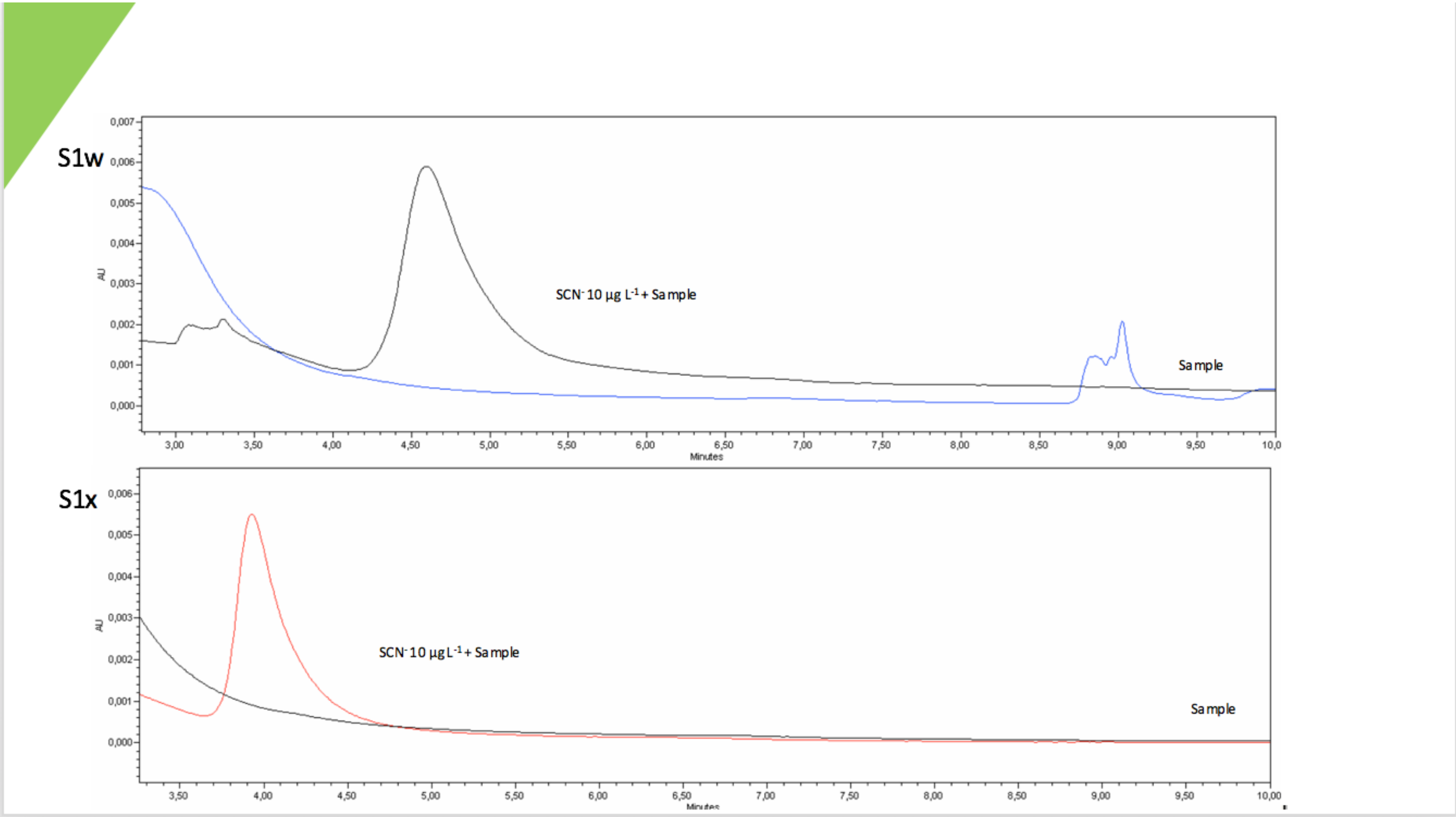
S1w
SCN- 10 µg L-1 + Sample
Sample
AU
Minutes
S1x
SCN- 10 µg L-1 + Sample
Sample
AU
Minutes

S1y

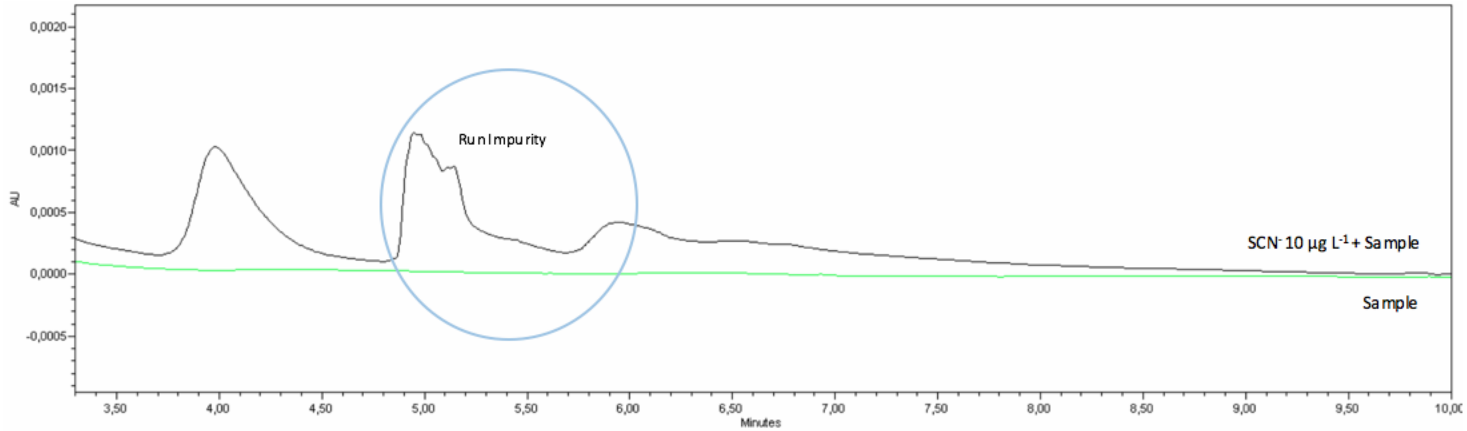
0,0020
0,0015
0,0010
0,0005
0,0000
-0,0005
AU
Run Impurity
SCN⁻ 10 µg L⁻¹ + Sample
Sample
3,50
4,00
4,50
5,00
5,50
6,00
6,50
7,00
7,50
8,00
8,50
9,00
9,50
10,0
Minutes

S1z

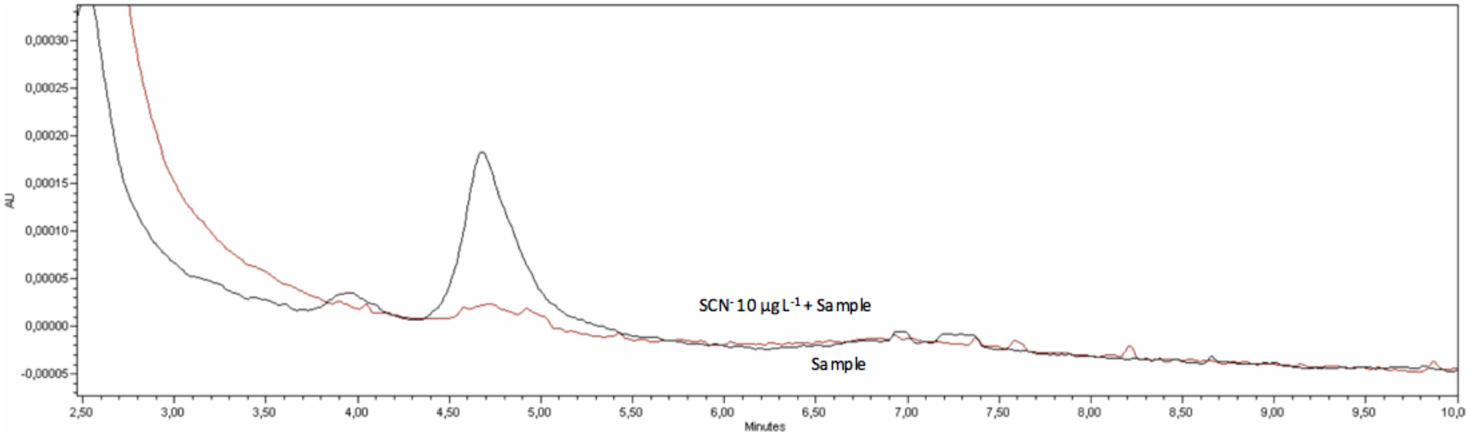
0,00030
0,00025
0,00020
0,00015
0,00010
0,00005
0,00000
-0,00005
AU
SCN⁻ 10 µg L⁻¹ + Sample
Sample
2,50
3,00
3,50
4,00
4,50
5,00
5,50
6,00
6,50
7,00
7,50
8,00
8,50
9,00
9,50
10,0
Minutes

S1aa

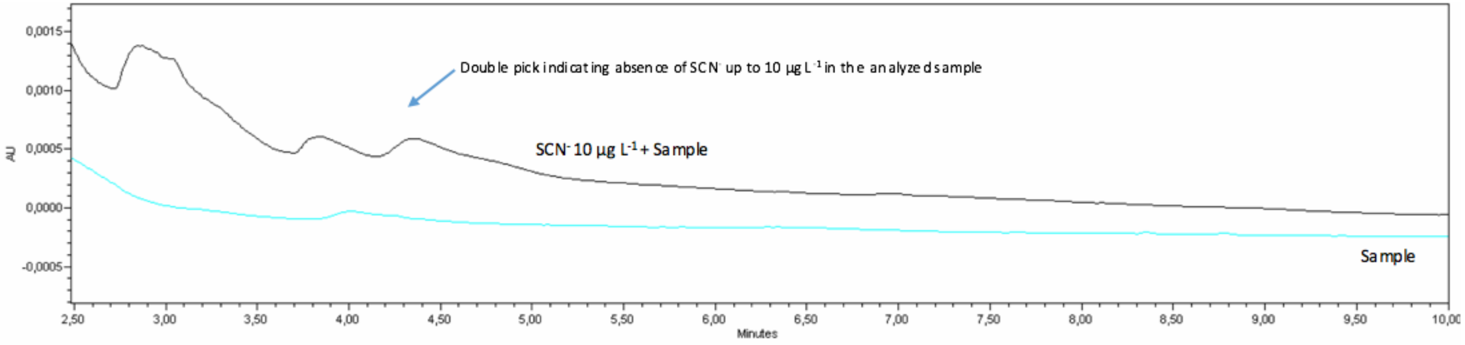
Double pick indicating absence of SCN- up to 10 µg L-1 in the analyzed sample
SCN- 10 µg L-1 + Sample
Sample
AU
Minutes

S1ab

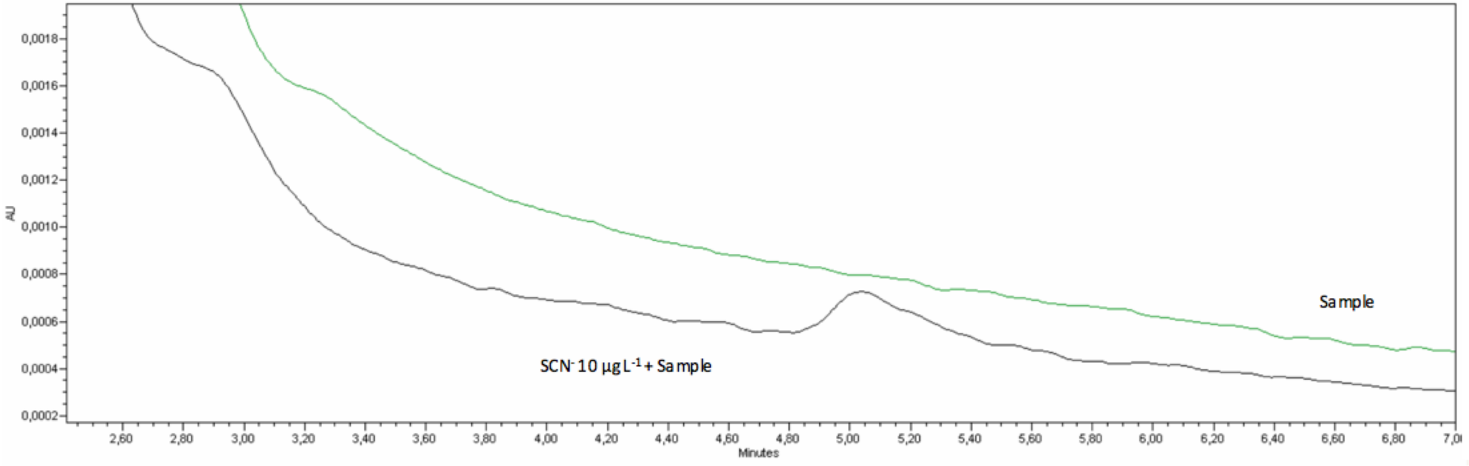
Sample
SCN- 10 µg L-1 + Sample
AU
Minutes

S1ac

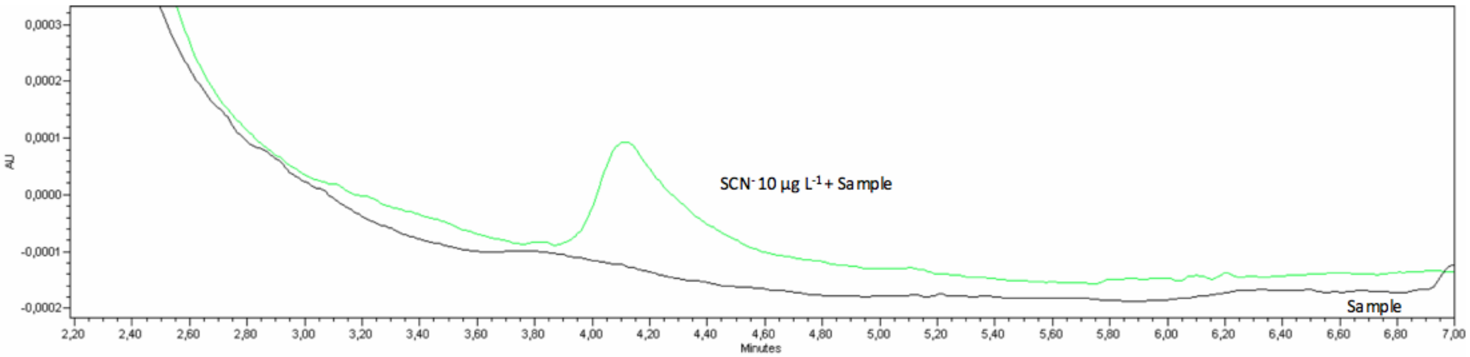

0,0003
0,0002
0,0001
0,0000
-0,0001
-0,0002
AU
SCN- 10 µg L-1 + Sample
Sample
2,20
2,40
2,60
2,80
3,00
3,20
3,40
3,60
3,80
4,00
4,20
4,40
4,60
4,80
5,00
5,20
5,40
5,60
5,80
6,00
6,20
6,40
6,60
6,80
7,00
Minutes

S1ad

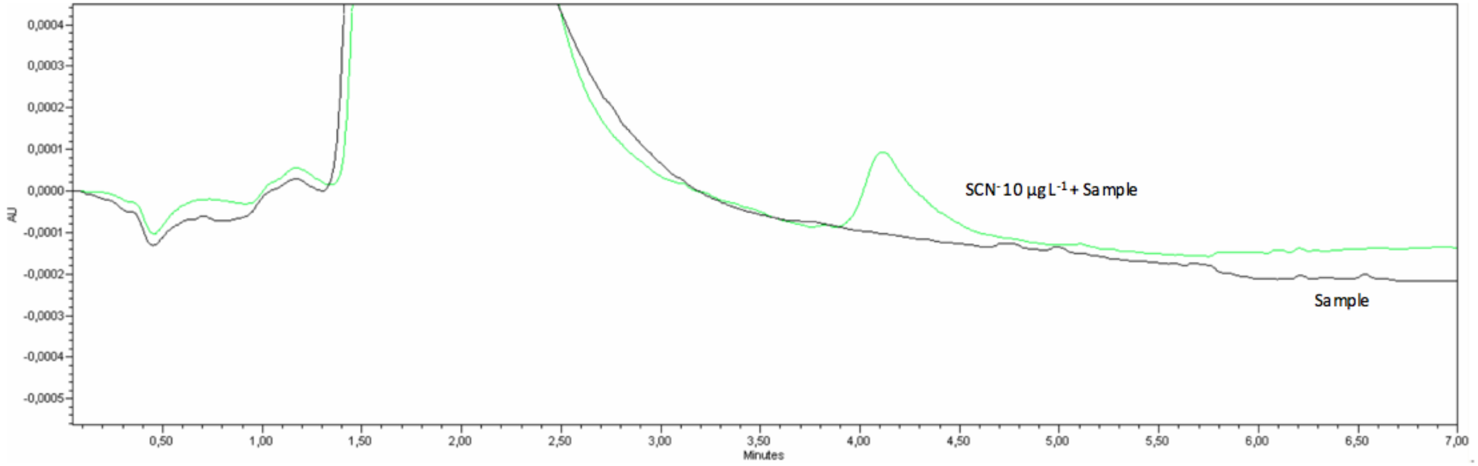

0,0004
0,0003
0,0002
0,0001
0,0000
-0,0001
-0,0002
-0,0003
-0,0004
-0,0005
AU
SCN- 10 µg L-1 + Sample
Sample
0,50
1,00
1,50
2,00
2,50
3,00
3,50
4,00
4,50
5,00
5,50
6,00
6,50
7,00
Minutes

S1ae

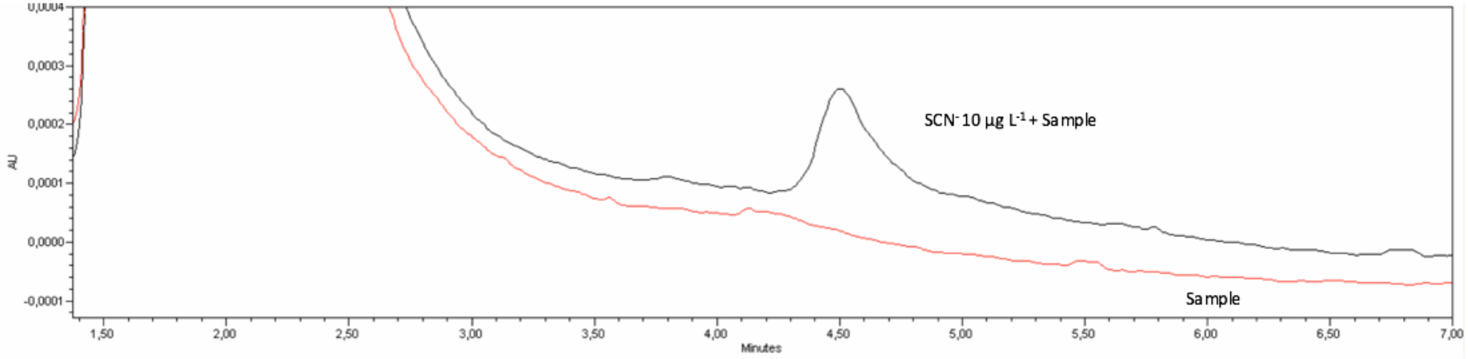
SCN- 10 µg L-1 + Sample
Sample
AU
Minutes

S1af

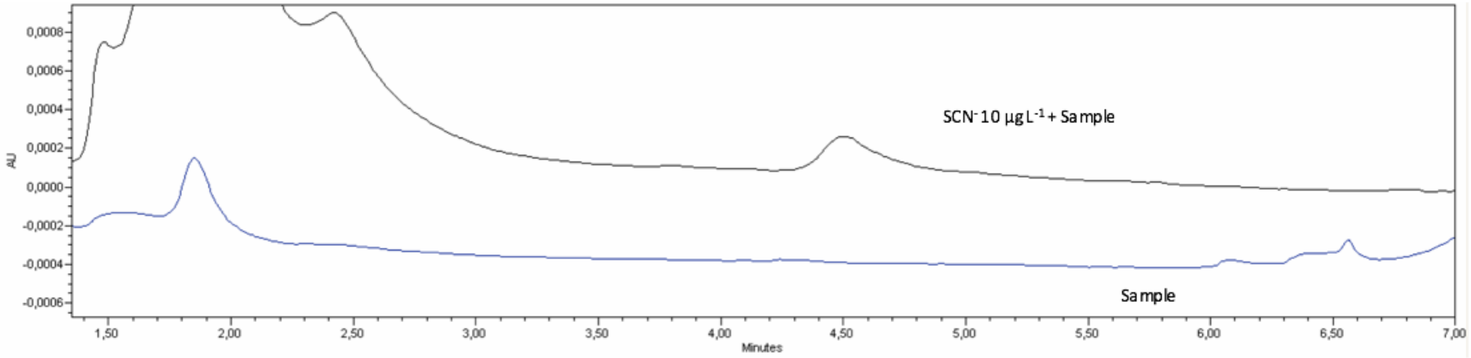
SCN- 10 µg L-1 + Sample
Sample
AU
Minutes

S1ag

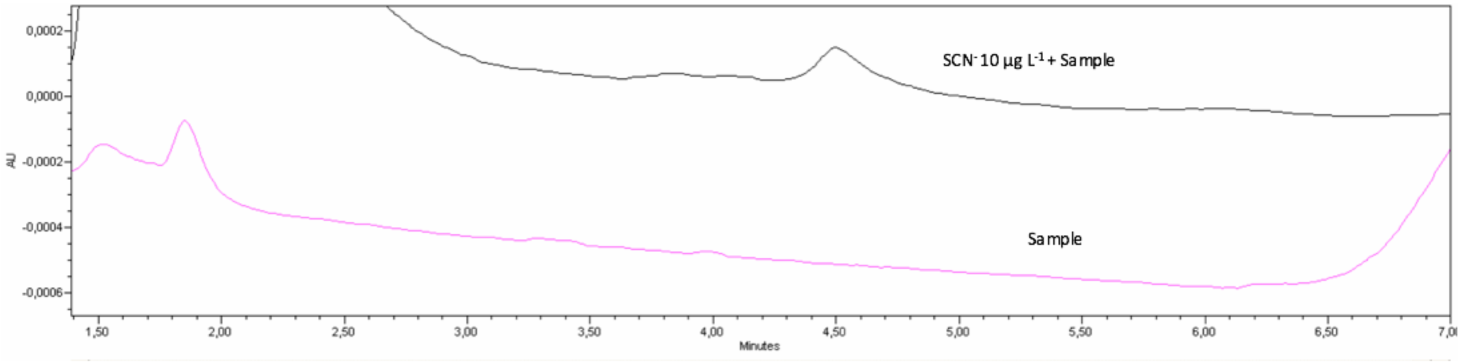
SCN^{-} 10 µg L^{-1} + Sample
Sample
AU
0,0002
0,0000
-0,0002
-0,0004
-0,0006
1,50
2,00
2,50
3,00
3,50
4,00
4,50
5,00
5,50
6,00
6,50
Minutes

S1ah

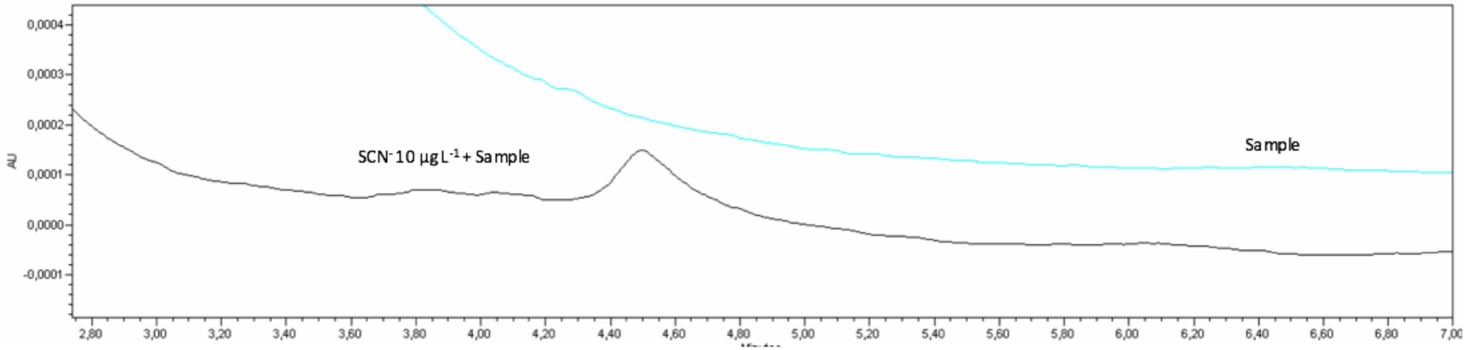
SCN^{-} 10 µg L^{-1} + Sample
Sample
AU
0,0004
0,0003
0,0002
0,0001
0,0000
-0,0001
2,80
3,00
3,20
3,40
3,60
3,80
4,00
4,20
4,40
4,60
4,80
5,00
5,20
5,40
5,60
5,80
6,00
6,20
6,40
6,60
6,80
7,00

S1ai

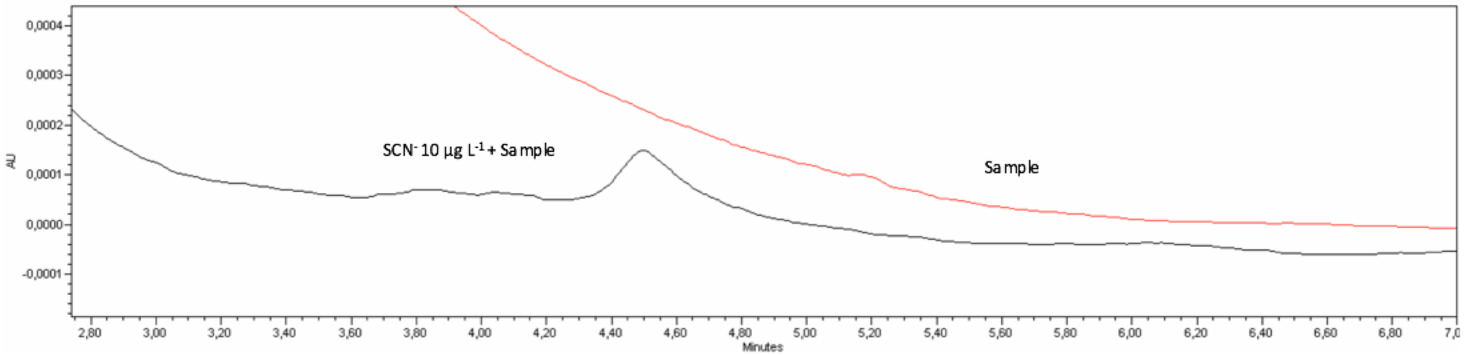
SCN⁻ 10 µg L⁻¹ + Sample
Sample
AU
Minutes

S1aj

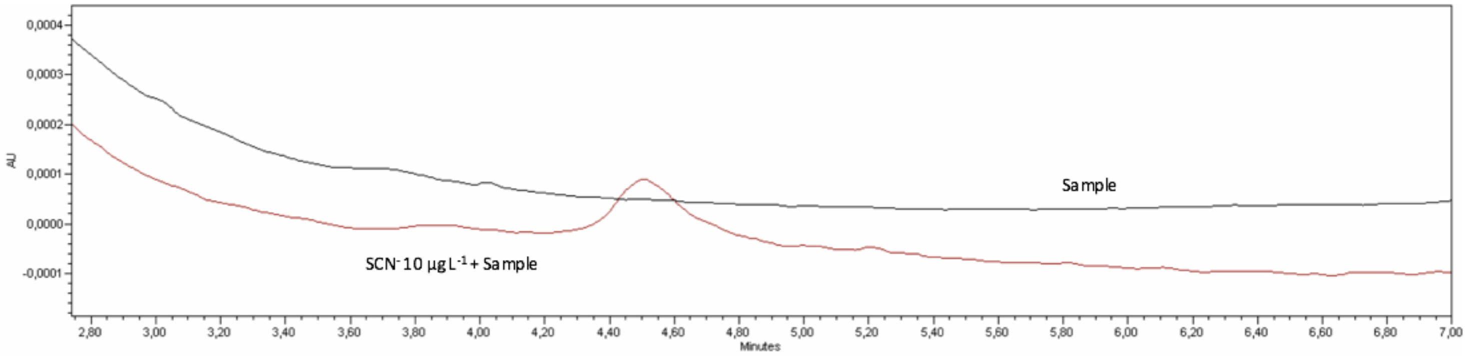
Sample
SCN⁻ 10 µg L⁻¹ + Sample
AU
Minutes

S1ak

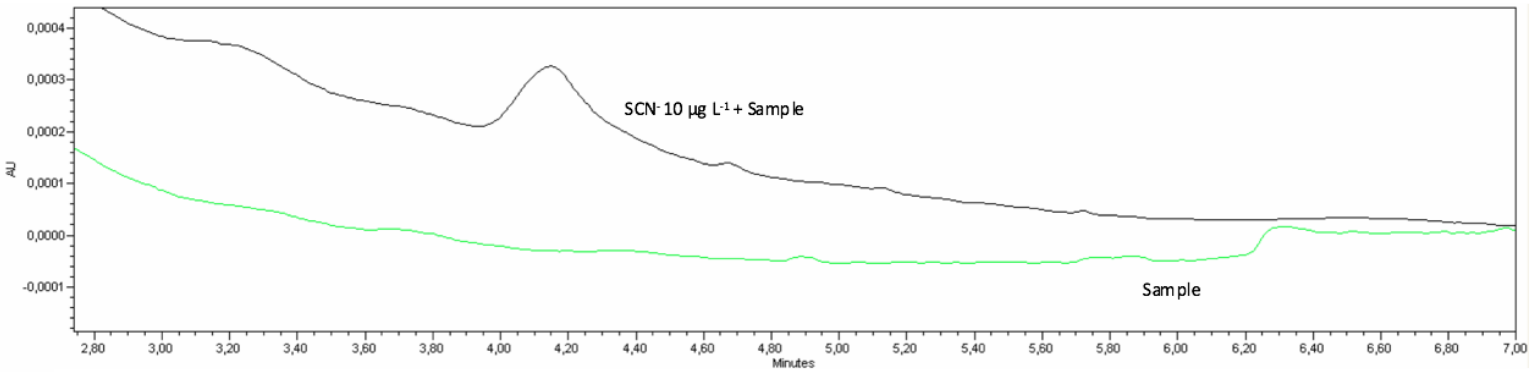
SCN- 10 µg L-1 + Sample
Sample
AU
0,0004
0,0003
0,0002
0,0001
0,0000
-0,0001
2,80 3,00 3,20 3,40 3,60 3,80 4,00 4,20 4,40 4,60 4,80 5,00 5,20 5,40 5,60 5,80 6,00 6,20 6,40 6,60 6,80 7,00
Minutes

S1al

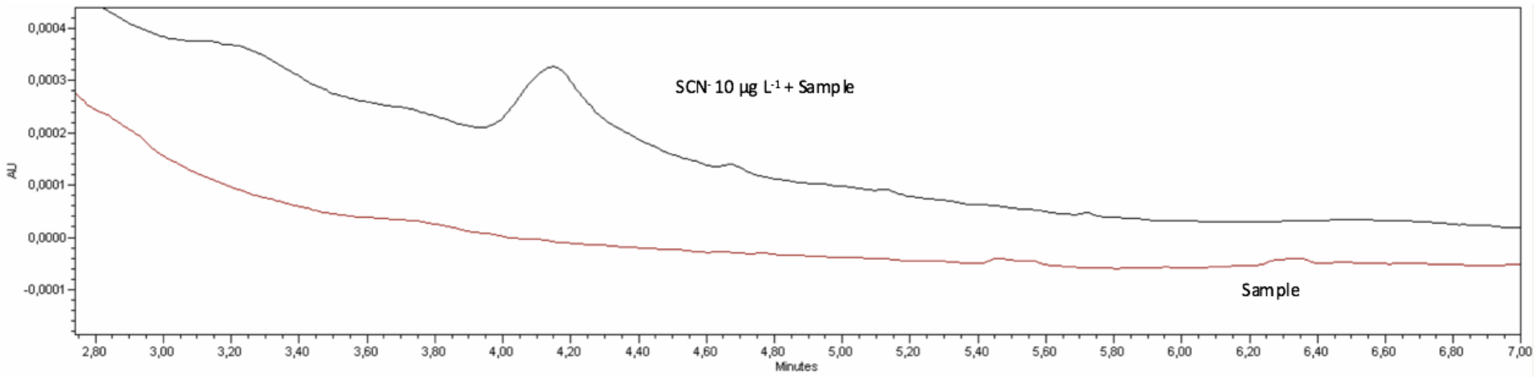
SCN- 10 µg L-1 + Sample
Sample
AU
0,0004
0,0003
0,0002
0,0001
0,0000
-0,0001
2,80 3,00 3,20 3,40 3,60 3,80 4,00 4,20 4,40 4,60 4,80 5,00 5,20 5,40 5,60 5,80 6,00 6,20 6,40 6,60 6,80 7,00
Minutes

S1am

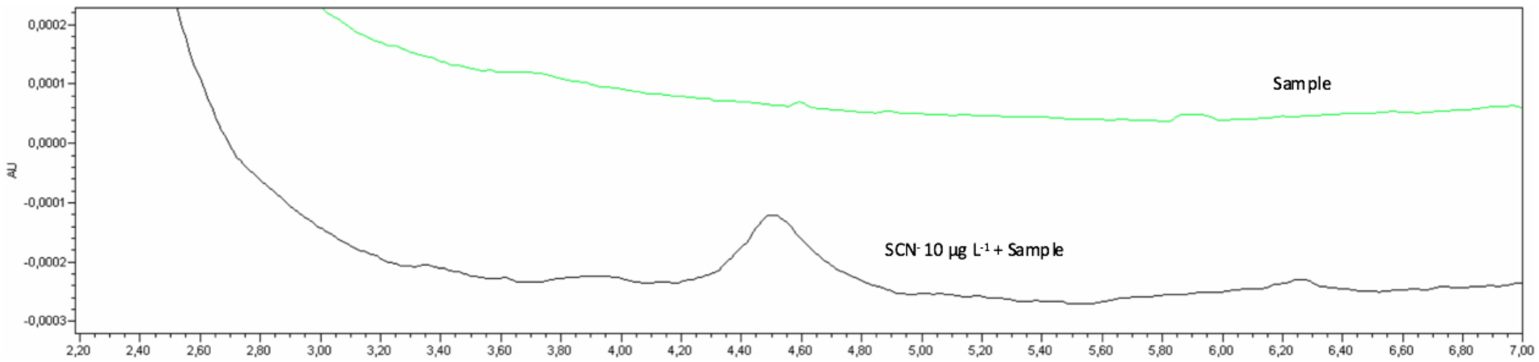
Sample
SCN- 10 µg L-1 + Sample
AU
0,0002
0,0001
0,0000
-0,0001
-0,0002
-0,0003
2,20 2,40 2,60 2,80 3,00 3,20 3,40 3,60 3,80 4,00 4,20 4,40 4,60 4,80 5,00 5,20 5,40 5,60 5,80 6,00 6,20 6,40 6,60 6,80 7,00

S1an

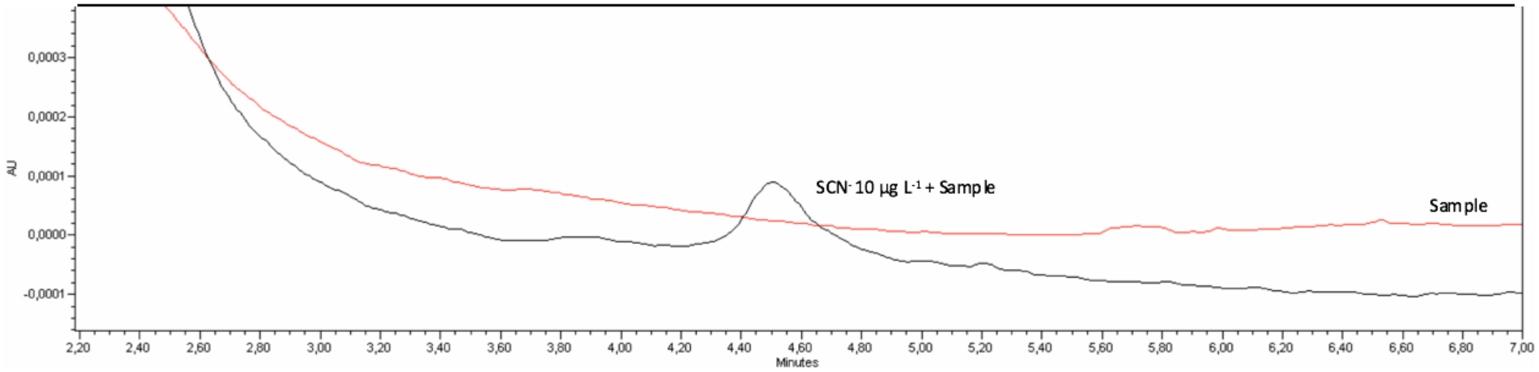
SCN- 10 µg L-1 + Sample
Sample
AU
0,0003
0,0002
0,0001
0,0000
-0,0001
2,20 2,40 2,60 2,80 3,00 3,20 3,40 3,60 3,80 4,00 4,20 4,40 4,60 4,80 5,00 5,20 5,40 5,60 5,80 6,00 6,20 6,40 6,60 6,80 7,00
Minutes

S1ao

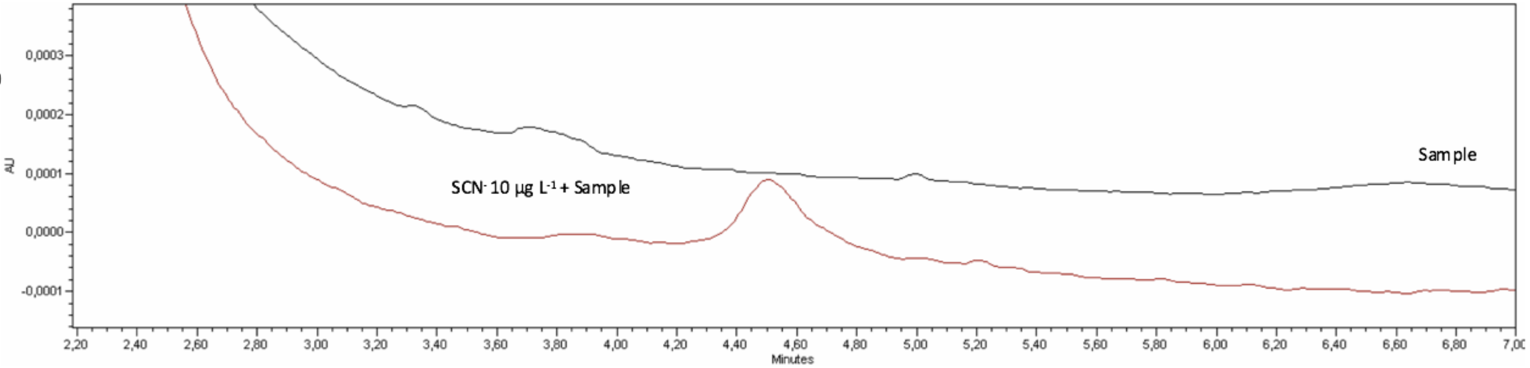
SCN- 10 µg L-1 + Sample
Sample
AU
0,0003
0,0002
0,0001
0,0000
-0,0001
2,20 2,40 2,60 2,80 3,00 3,20 3,40 3,60 3,80 4,00 4,20 4,40 4,60 4,80 5,00 5,20 5,40 5,60 5,80 6,00 6,20 6,40 6,60 6,80 7,00
Minutes

S1ap

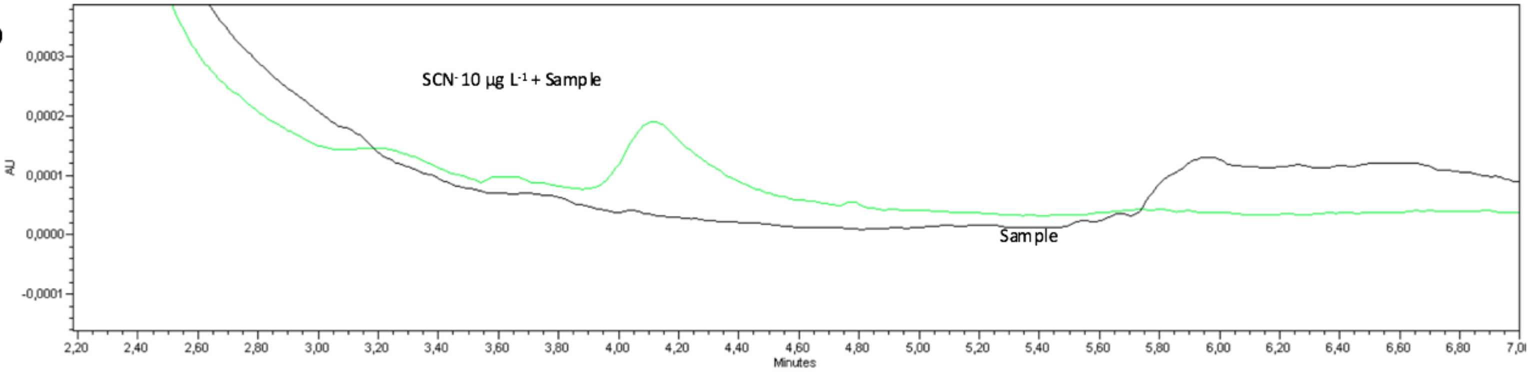
SCN- 10 µg L-1 + Sample
Sample
AU
0,0003
0,0002
0,0001
0,0000
-0,0001
2,20 2,40 2,60 2,80 3,00 3,20 3,40 3,60 3,80 4,00 4,20 4,40 4,60 4,80 5,00 5,20 5,40 5,60 5,80 6,00 6,20 6,40 6,60 6,80 7,00
Minutes

S1aq

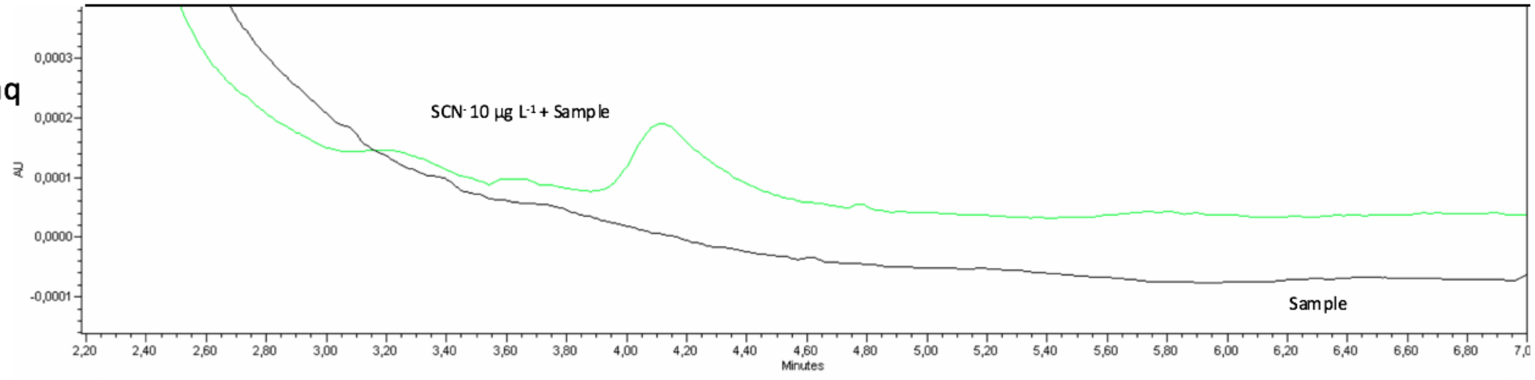
SCN- 10 µg L-1 + Sample
Sample
AU
0,0003
0,0002
0,0001
0,0000
-0,0001
2,20
2,40
2,60
2,80
3,00
3,20
3,40
3,60
3,80
4,00
4,20
4,40
4,60
4,80
5,00
5,20
5,40
5,60
5,80
6,00
6,20
6,40
6,60
6,80
7,0
Minutes

S1ar

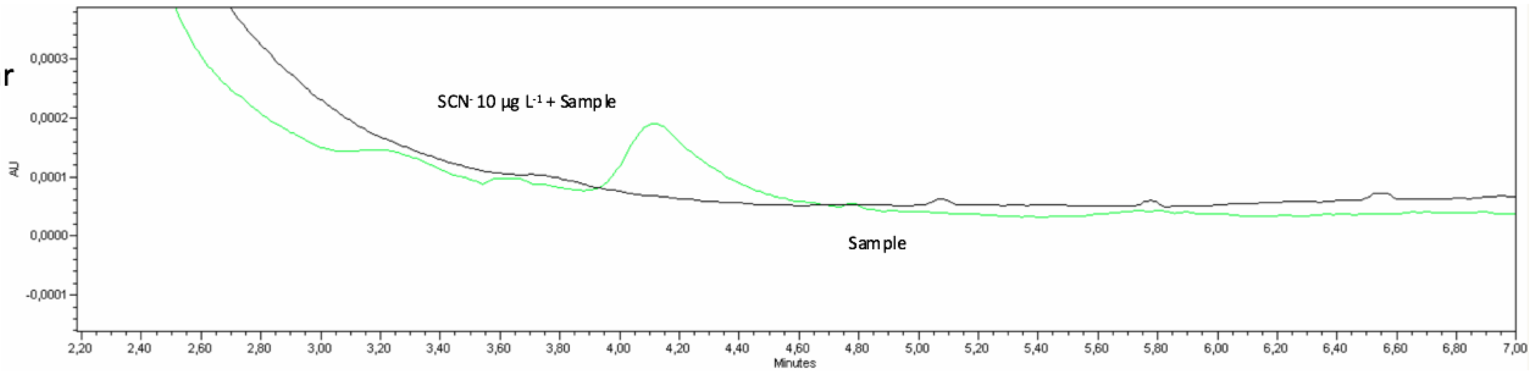
SCN- 10 µg L-1 + Sample
Sample
AU
0,0003
0,0002
0,0001
0,0000
-0,0001
2,20
2,40
2,60
2,80
3,00
3,20
3,40
3,60
3,80
4,00
4,20
4,40
4,60
4,80
5,00
5,20
5,40
5,60
5,80
6,00
6,20
6,40
6,60
6,80
7,00
Minutes

S1as

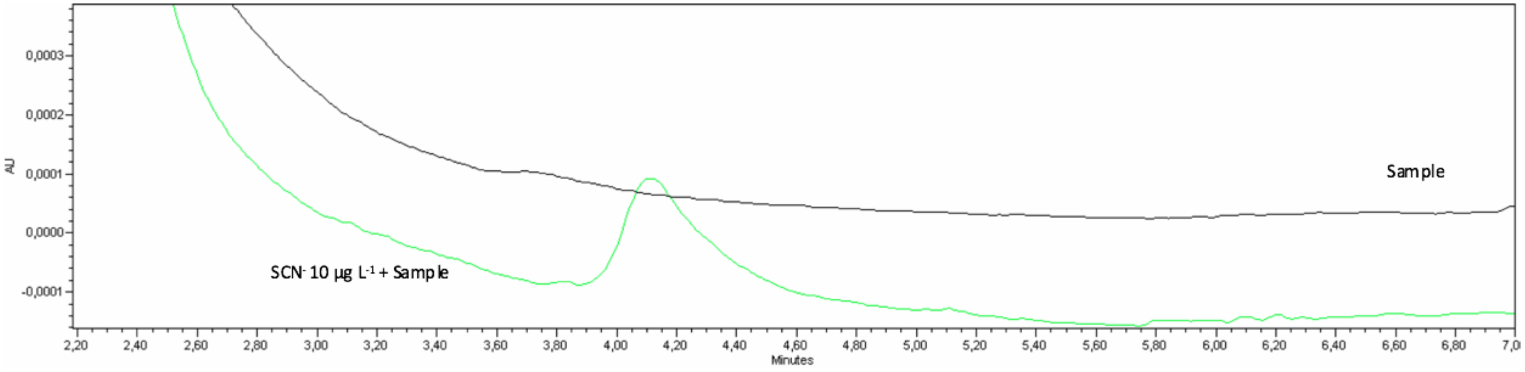

Sample
SCN- 10 µg L-1 + Sample
AU
0,0003
0,0002
0,0001
0,0000
-0,0001
2,20 2,40 2,60 2,80 3,00 3,20 3,40 3,60 3,80 4,00 4,20 4,40 4,60 4,80 5,00 5,20 5,40 5,60 5,80 6,00 6,20 6,40 6,60 6,80
Minutes

S1at

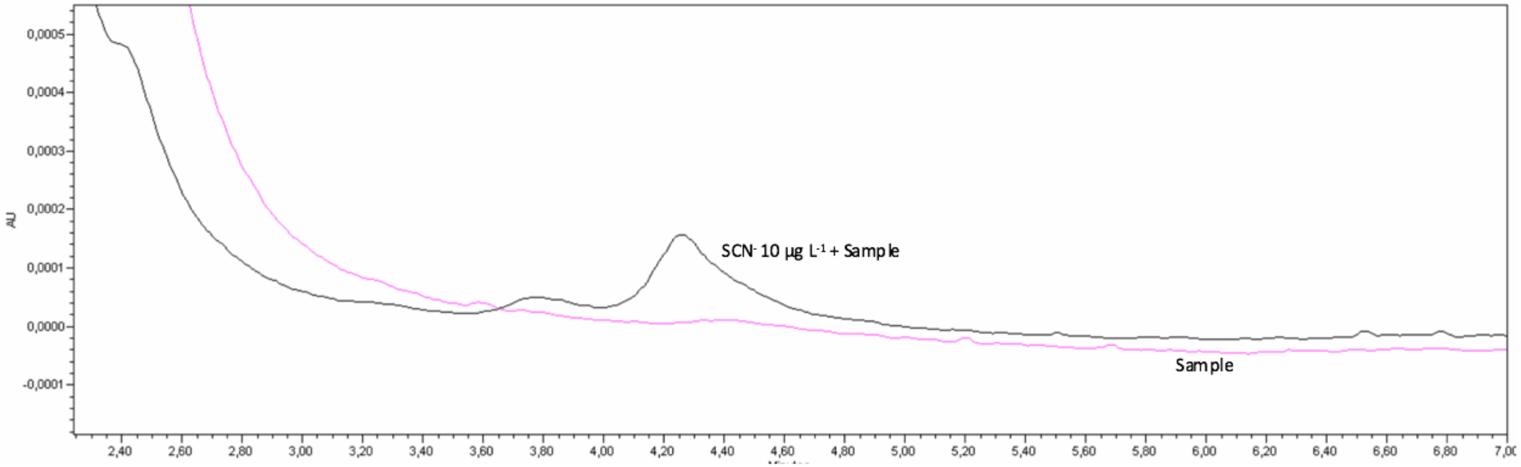

SCN- 10 µg L-1 + Sample
Sample
AU
0,0005
0,0004
0,0003
0,0002
0,0001
0,0000
-0,0001
2,40 2,60 2,80 3,00 3,20 3,40 3,60 3,80 4,00 4,20 4,40 4,60 4,80 5,00 5,20 5,40 5,60 5,80 6,00 6,20 6,40 6,60 6,80

S1au

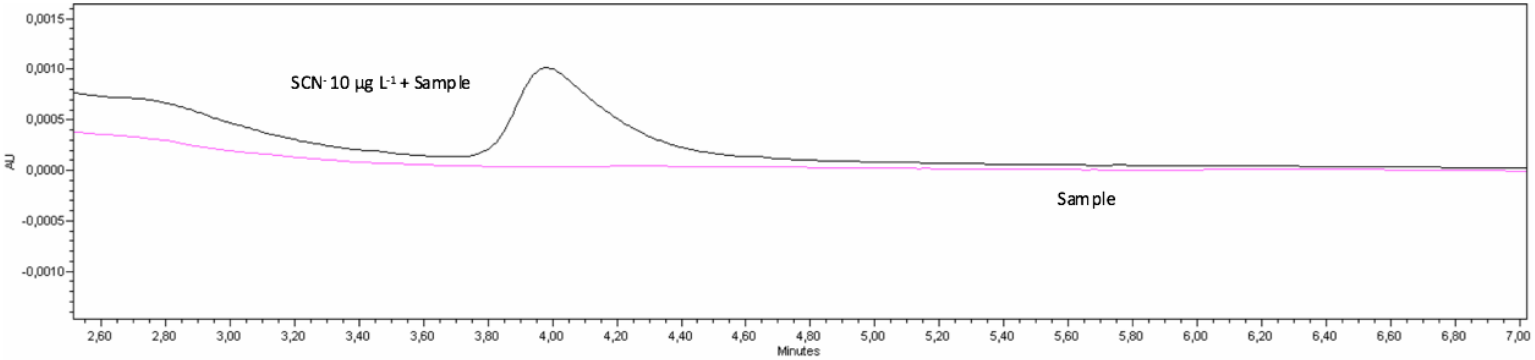
SCN- 10 µg L-1 + Sample
Sample
AU
Minutes

S1av

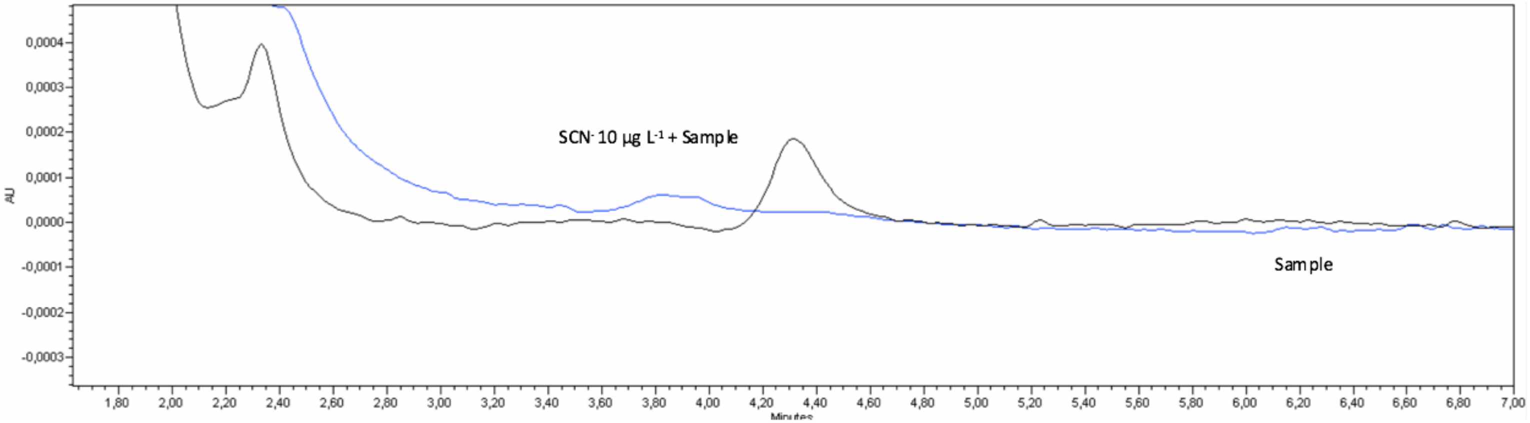
SCN- 10 µg L-1 + Sample
Sample
AU

S1aw

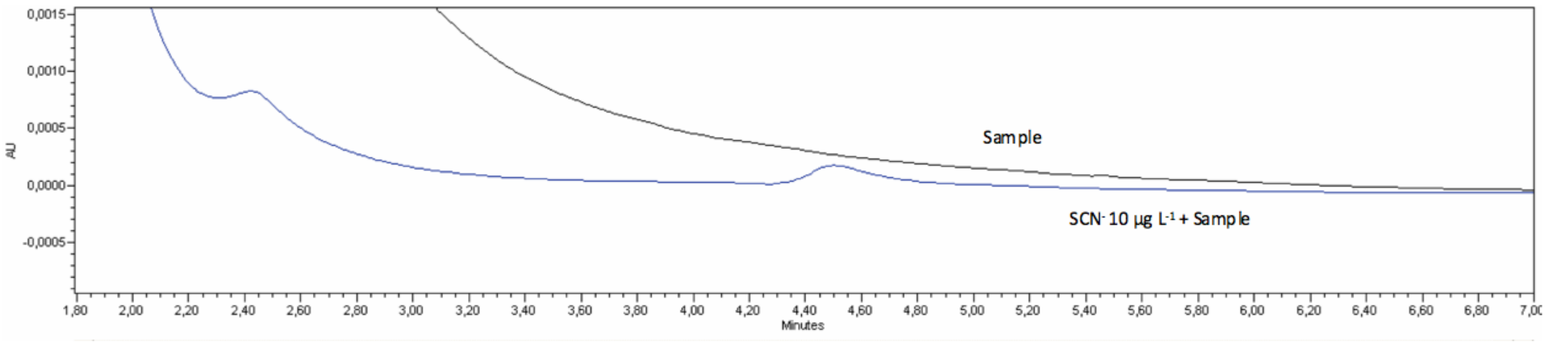

0,0015
0,0010
0,0005
0,0000
-0,0005
AU
1,80 2,00 2,20 2,40 2,60 2,80 3,00 3,20 3,40 3,60 3,80 4,00 4,20 4,40 4,60 4,80 5,00 5,20 5,40 5,60 5,80 6,00 6,20 6,40 6,60 6,80
Minutes
Sample
SCN- 10 µg L-1 + Sample

S1ax

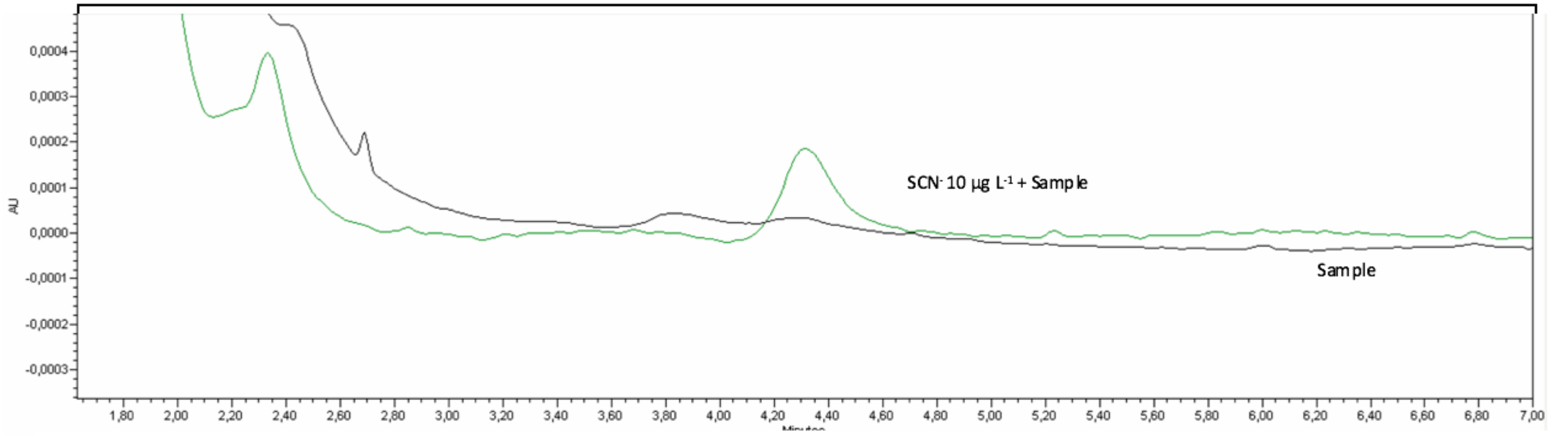

0,0004
0,0003
0,0002
0,0001
0,0000
-0,0001
-0,0002
-0,0003
AU
1,80 2,00 2,20 2,40 2,60 2,80 3,00 3,20 3,40 3,60 3,80 4,00 4,20 4,40 4,60 4,80 5,00 5,20 5,40 5,60 5,80 6,00 6,20 6,40 6,60 6,80 7,00
SCN- 10 µg L-1 + Sample
Sample

S1ay

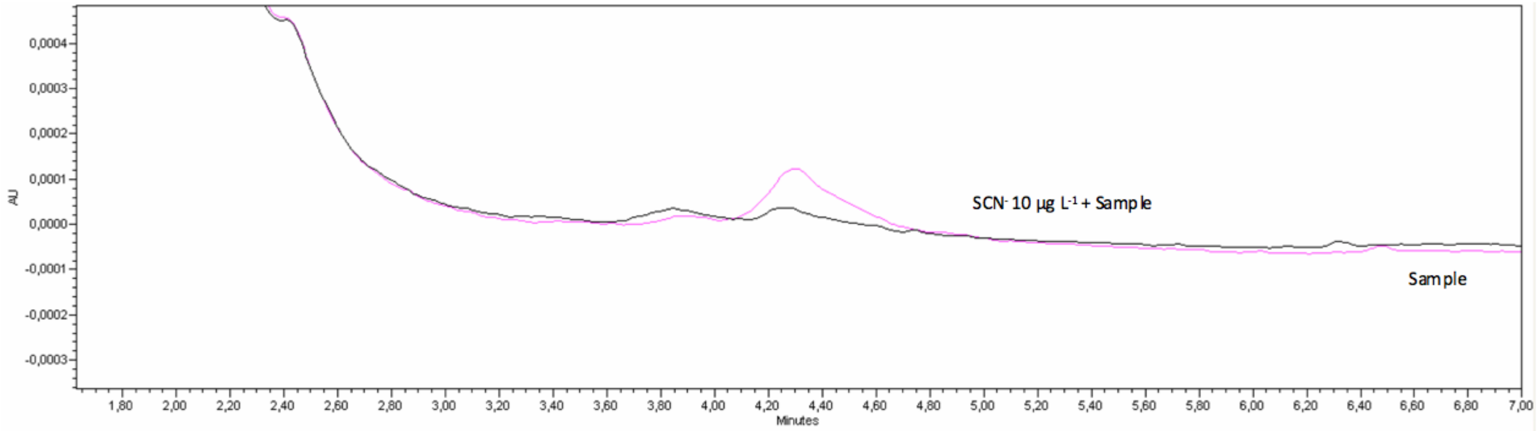
AU
0,0004
0,0003
0,0002
0,0001
0,0000
-0,0001
-0,0002
-0,0003
1,80 2,00 2,20 2,40 2,60 2,80 3,00 3,20 3,40 3,60 3,80 4,00 4,20 4,40 4,60 4,80 5,00 5,20 5,40 5,60 5,80 6,00 6,20 6,40 6,60 6,80 7,00
Minutes
SCN^- 10 µg L^{-1} + Sample
Sample

S1az

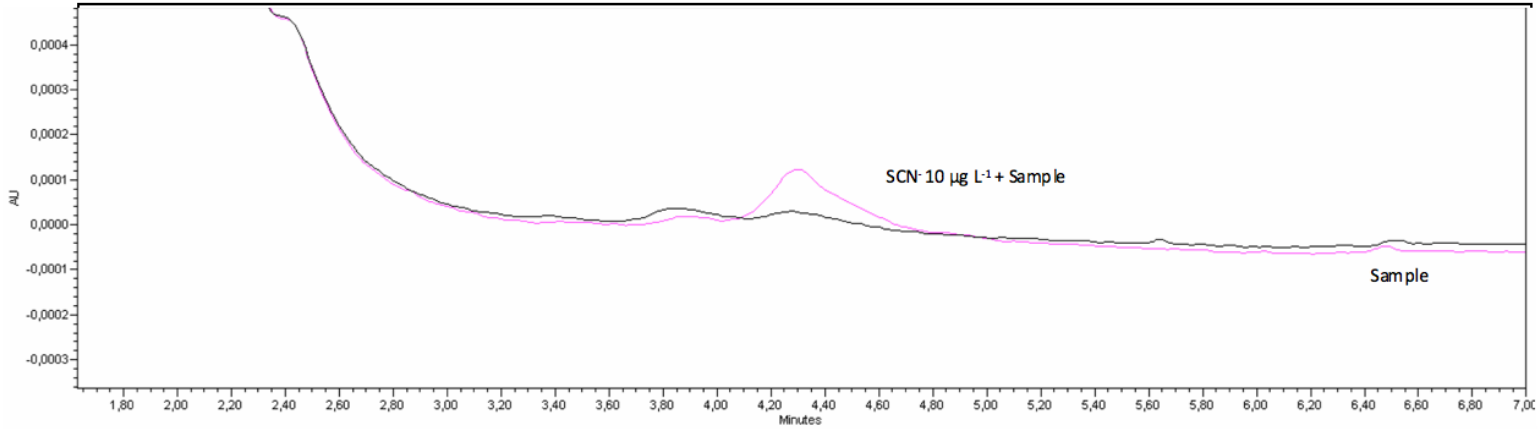
AU
0,0004
0,0003
0,0002
0,0001
0,0000
-0,0001
-0,0002
-0,0003
1,80 2,00 2,20 2,40 2,60 2,80 3,00 3,20 3,40 3,60 3,80 4,00 4,20 4,40 4,60 4,80 5,00 5,20 5,40 5,60 5,80 6,00 6,20 6,40 6,60 6,80 7,00
Minutes
SCN^- 10 µg L^{-1} + Sample
Sample

S1ba

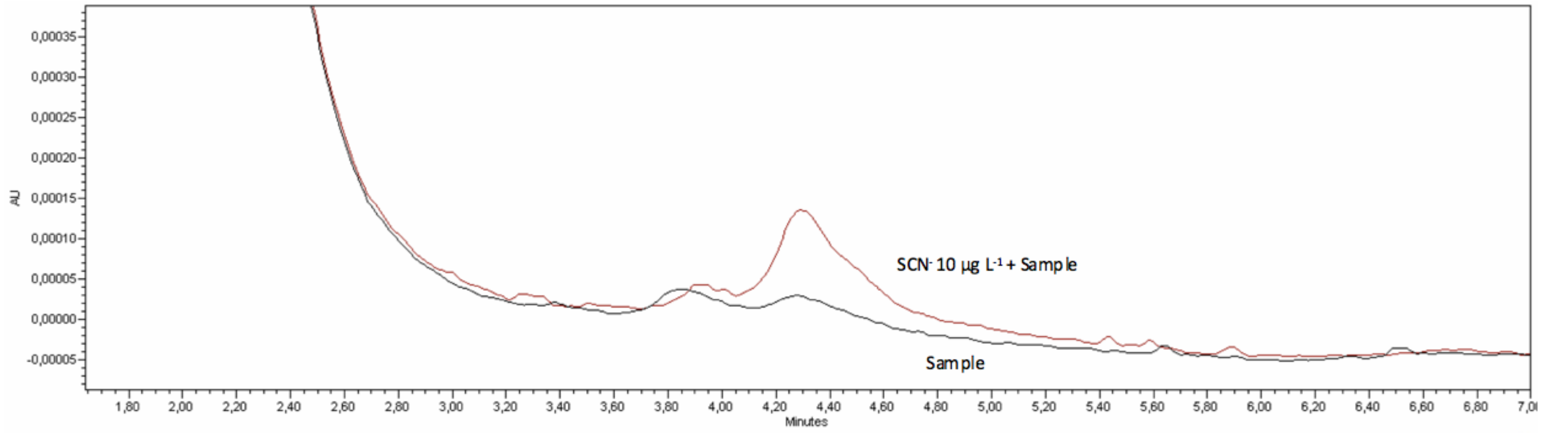
AU
Minutes
SCN- 10 µg L-1 + Sample
Sample

S1bb

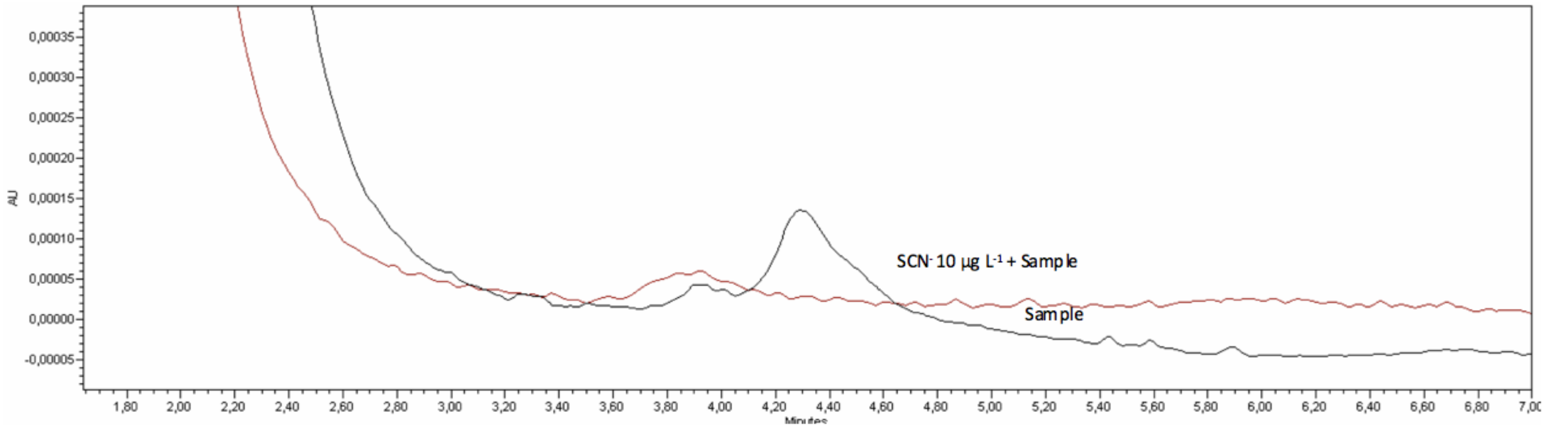
AU
SCN- 10 µg L-1 + Sample
Sample

S1bc

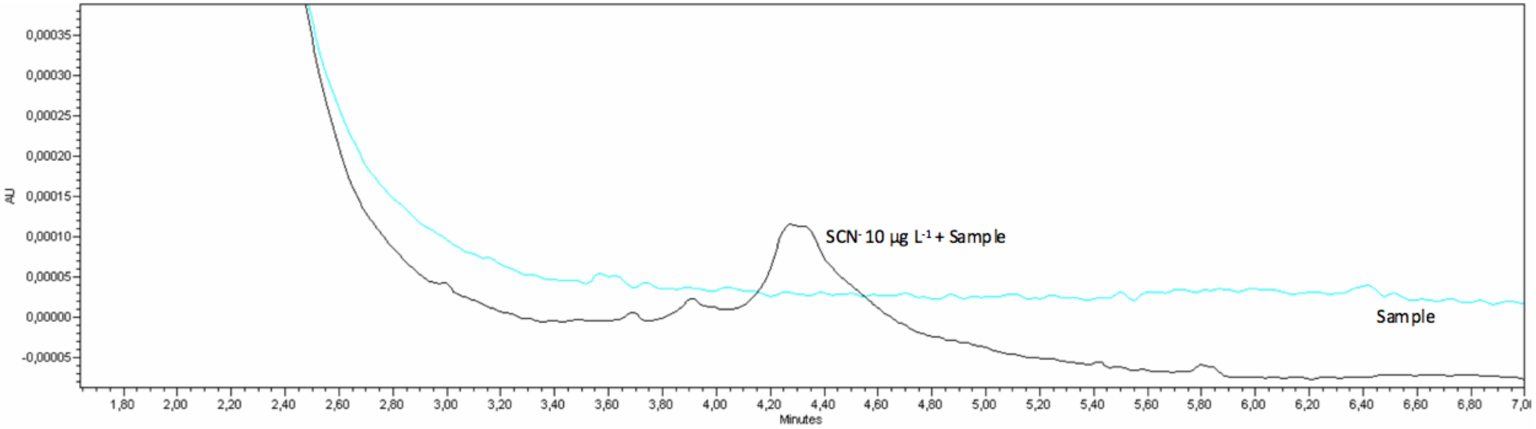
SCN- 10 µg L-1 + Sample
Sample
AU
Minutes

S1bd

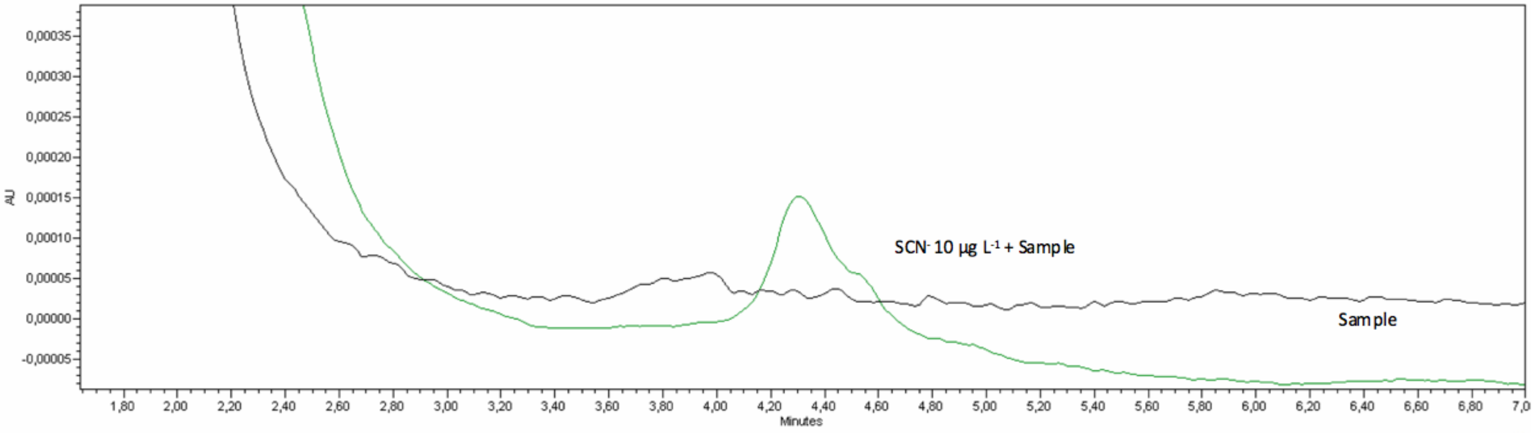
SCN- 10 µg L-1 + Sample
Sample
AU
Minutes

S1be

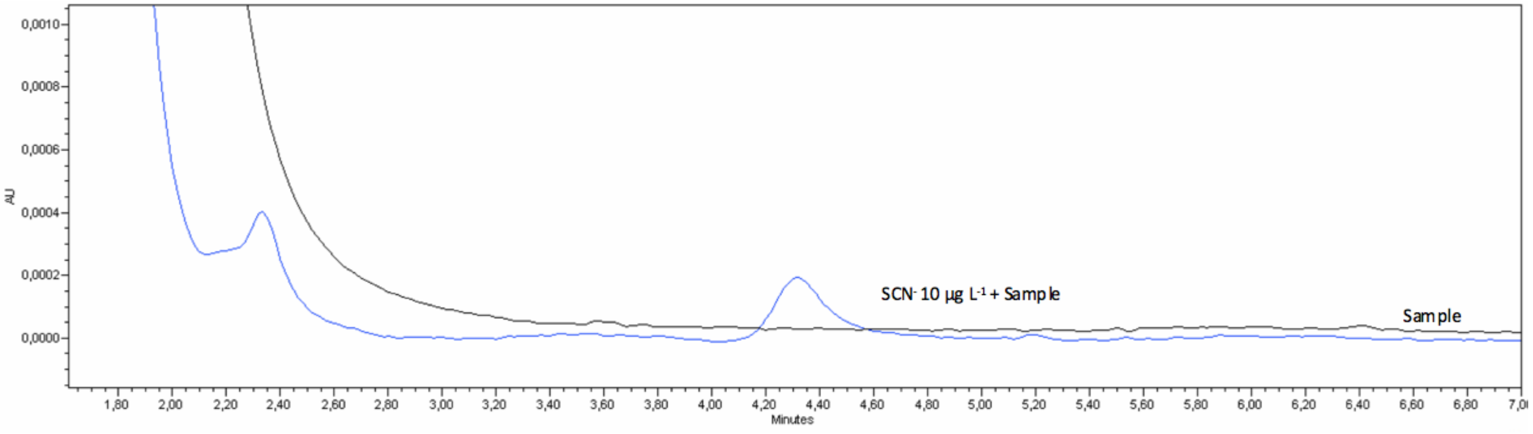

0,0010
0,0008
0,0006
0,0004
0,0002
0,0000
AU
1,80 2,00 2,20 2,40 2,60 2,80 3,00 3,20 3,40 3,60 3,80 4,00 4,20 4,40 4,60 4,80 5,00 5,20 5,40 5,60 5,80 6,00 6,20 6,40 6,60 6,80
Minutes
SCN- 10 µg L-1 + Sample
Sample

S1bf

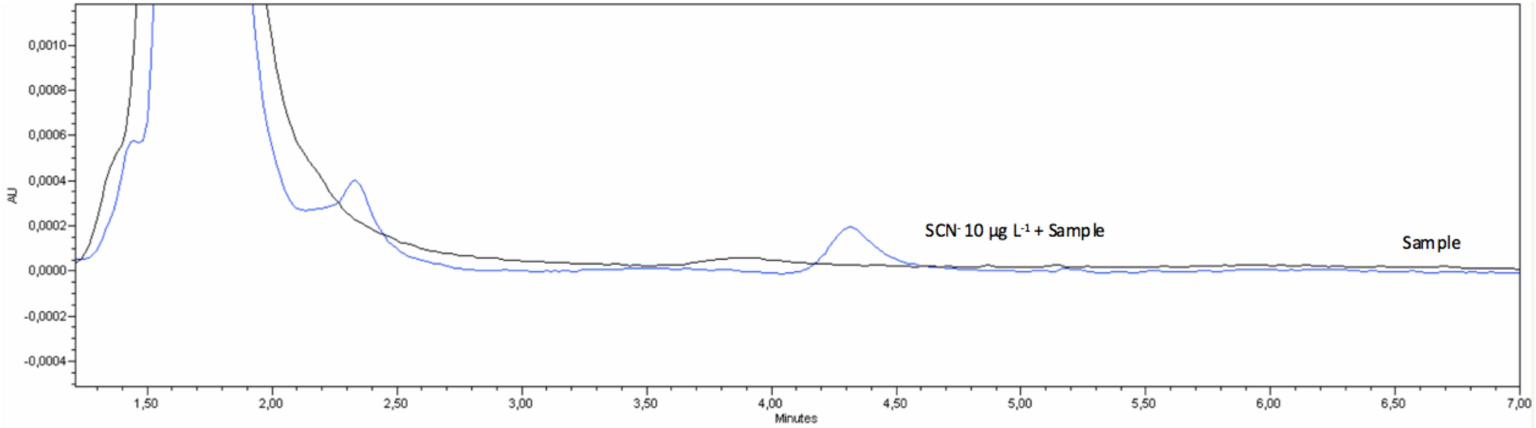

0,0010
0,0008
0,0006
0,0004
0,0002
0,0000
-0,0002
-0,0004
AU
1,50 2,00 2,50 3,00 3,50 4,00 4,50 5,00 5,50 6,00 6,50 7,00
Minutes
SCN- 10 µg L-1 + Sample
Sample

S1bg

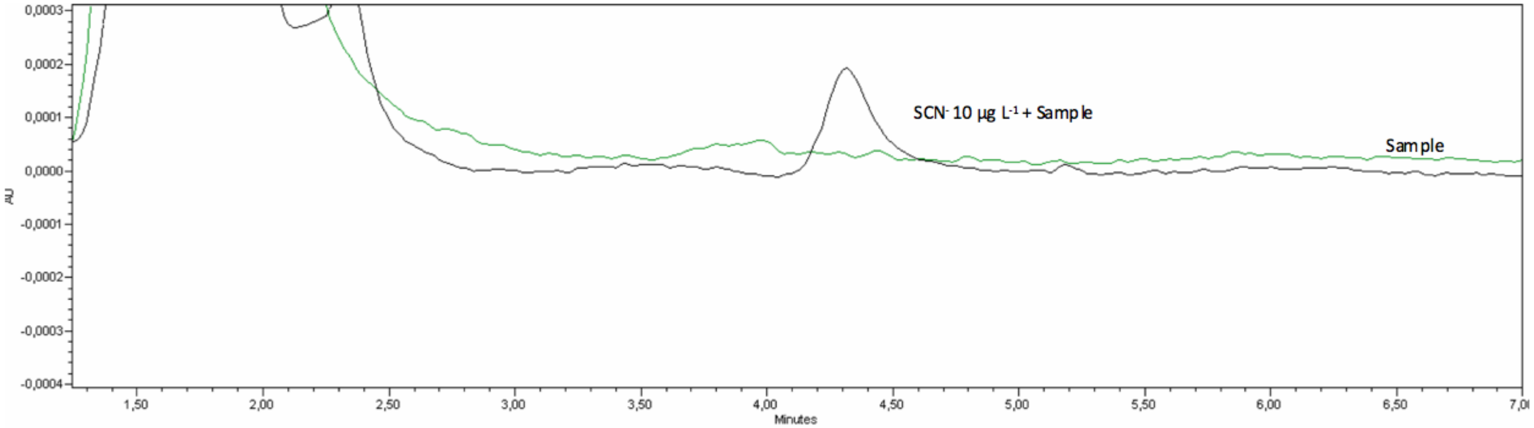
SCN- 10 µg L-1 + Sample
Sample
AU
Minutes
0,0003
0,0002
0,0001
0,0000
-0,0001
-0,0002
-0,0003
-0,0004
1,50
2,00
2,50
3,00
3,50
4,00
4,50
5,00
5,50
6,00
6,50

S1bh

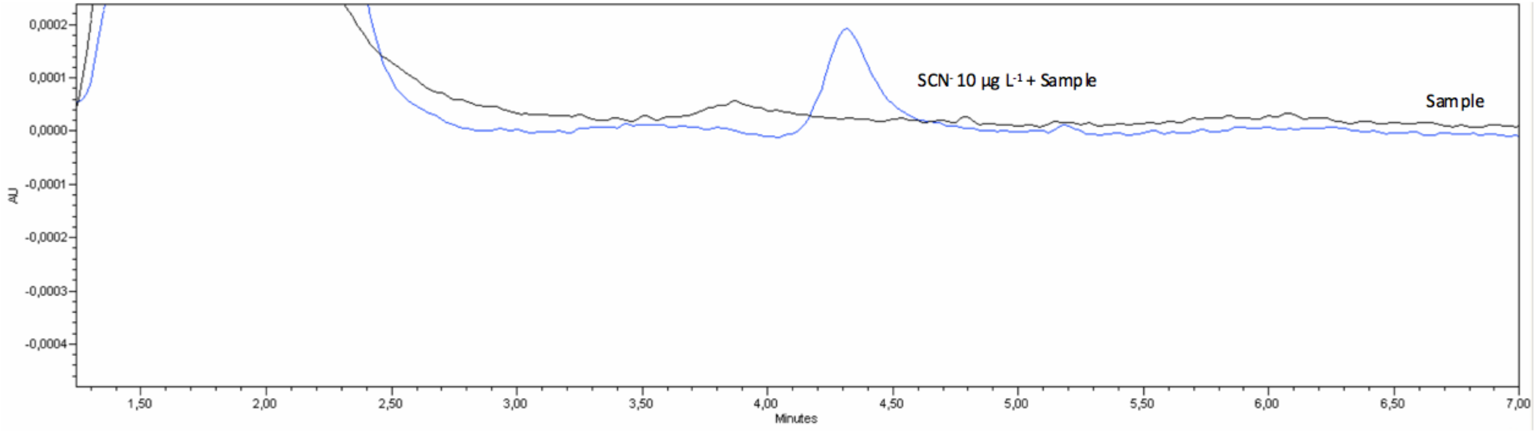
SCN- 10 µg L-1 + Sample
Sample
AU
Minutes
0,0002
0,0001
0,0000
-0,0001
-0,0002
-0,0003
-0,0004
1,50
2,00
2,50
3,00
3,50
4,00
4,50
5,00
5,50
6,00
6,50
7,00

S1bi

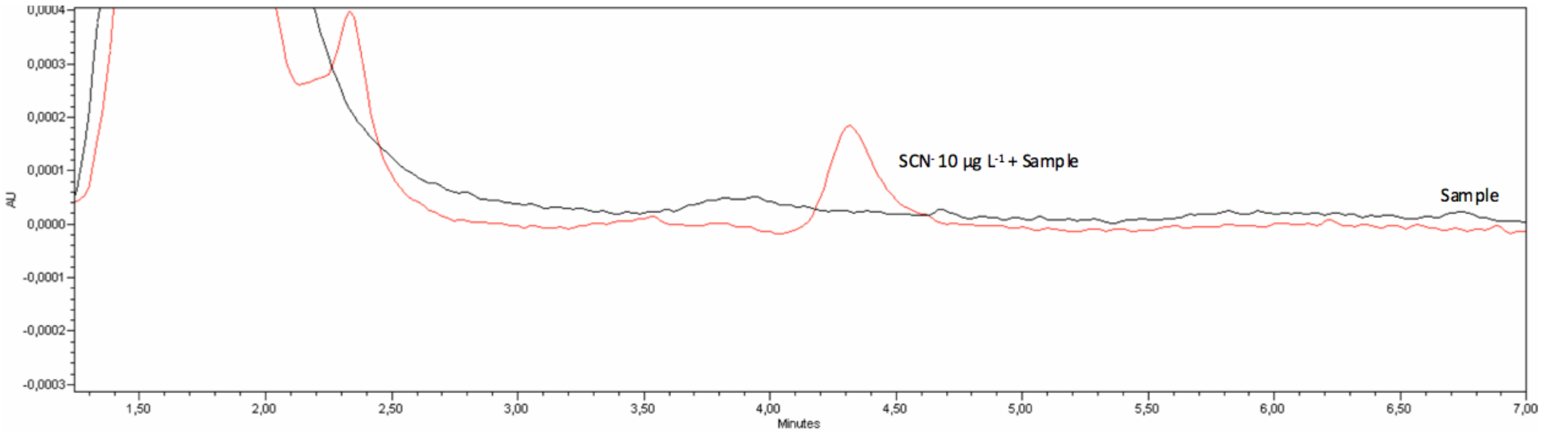
SCN- 10 µg L-1 + Sample
Sample
AU
0,0004
0,0003
0,0002
0,0001
0,0000
-0,0001
-0,0002
-0,0003
1,50
2,00
2,50
3,00
3,50
4,00
4,50
5,00
5,50
6,00
6,50
7,00
Minutes

S1bj

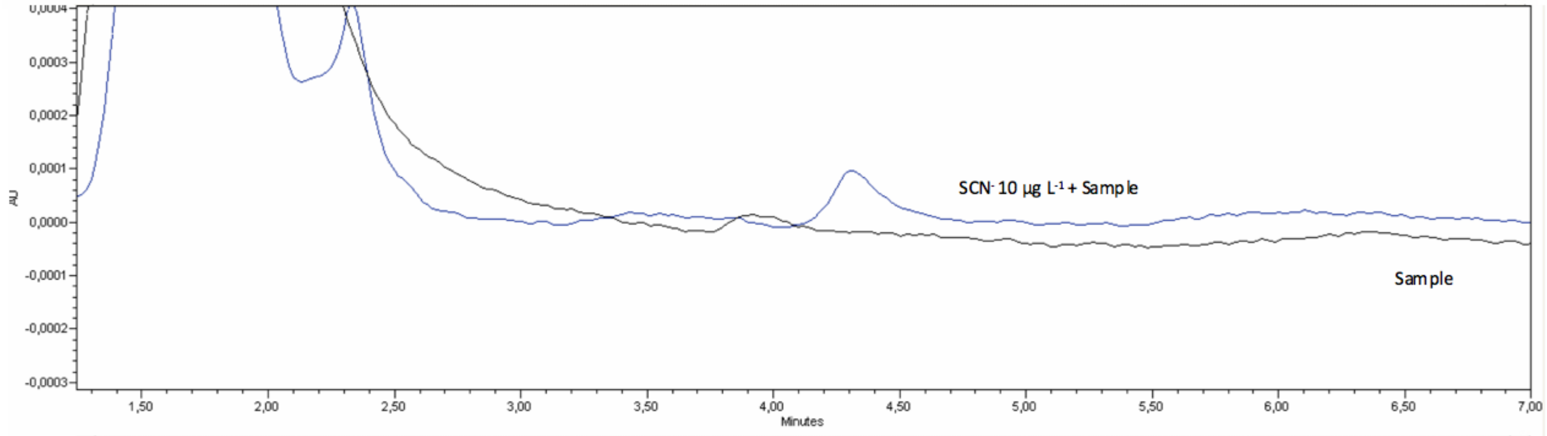
SCN- 10 µg L-1 + Sample
Sample
AU
0,0004
0,0003
0,0002
0,0001
0,0000
-0,0001
-0,0002
-0,0003
1,50
2,00
2,50
3,00
3,50
4,00
4,50
5,00
5,50
6,00
6,50
7,00
Minutes

S1bk

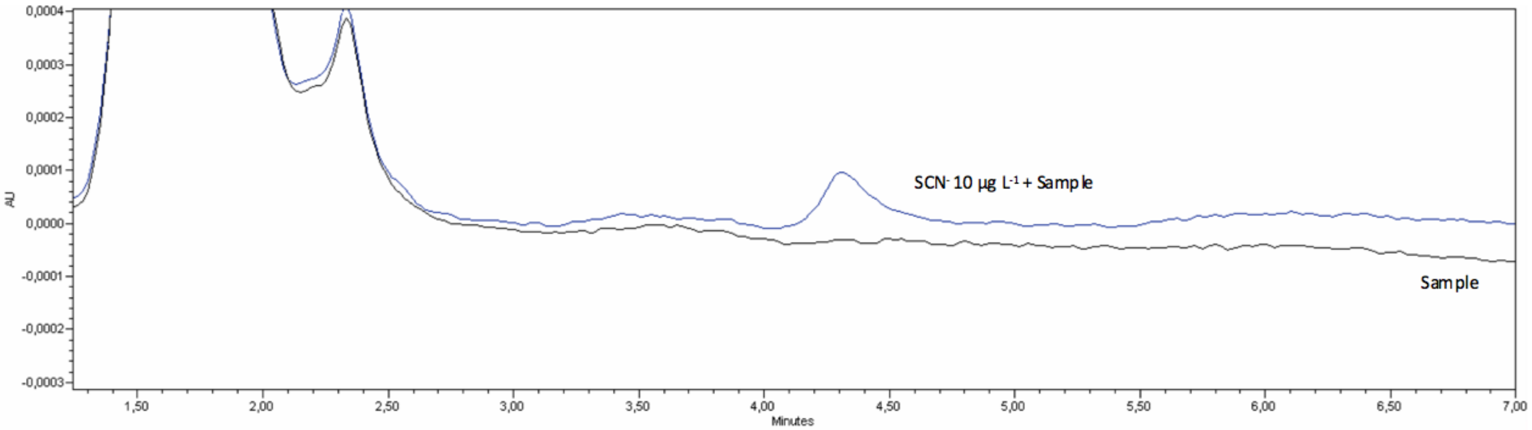
0,0004
0,0003
0,0002
0,0001
0,0000
-0,0001
-0,0002
-0,0003
AU
1,50
2,00
2,50
3,00
3,50
4,00
4,50
5,00
5,50
6,00
6,50
7,00
Minutes
SCN- 10 µg L-1 + Sample
Sample

S1bl

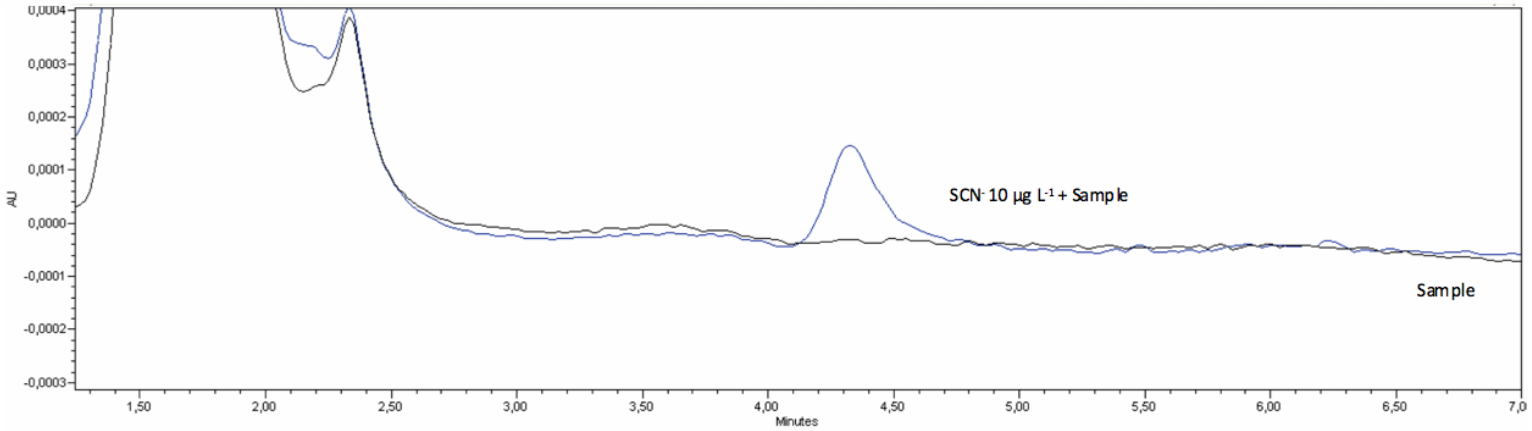
0,0004
0,0003
0,0002
0,0001
0,0000
-0,0001
-0,0002
-0,0003
AU
1,50
2,00
2,50
3,00
3,50
4,00
4,50
5,00
5,50
6,00
6,50
7,0
Minutes
SCN- 10 µg L-1 + Sample
Sample

S1bm

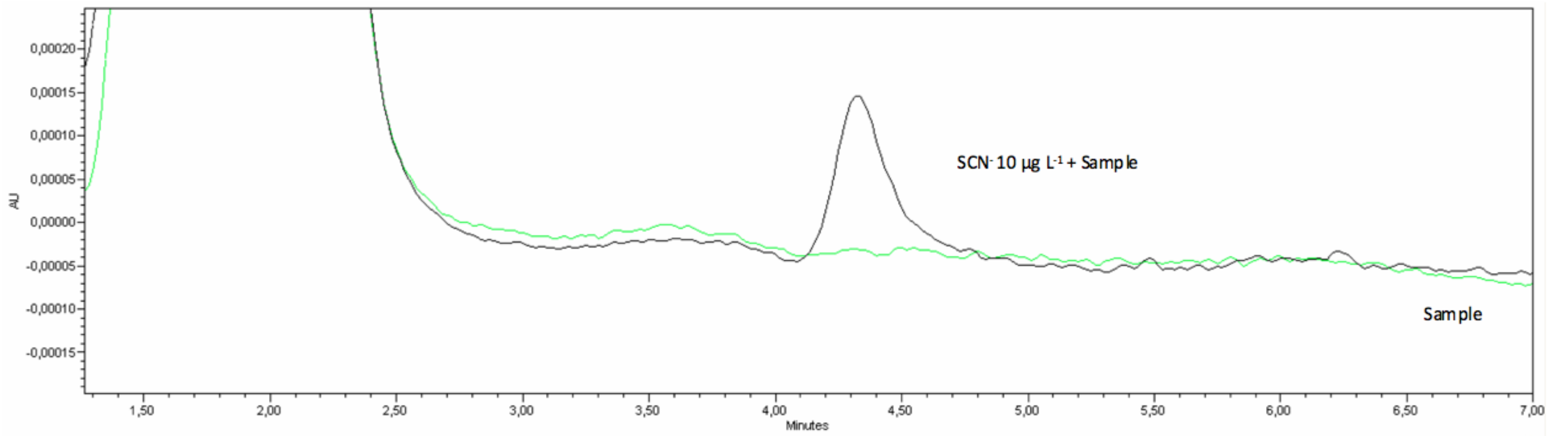
SCN- 10 µg L-1 + Sample
Sample
AU
Minutes
0,00020
0,00015
0,00010
0,00005
0,00000
-0,00005
-0,00010
-0,00015
1,50
2,00
2,50
3,00
3,50
4,00
4,50
5,00
5,50
6,00
6,50
7,00

S1bn

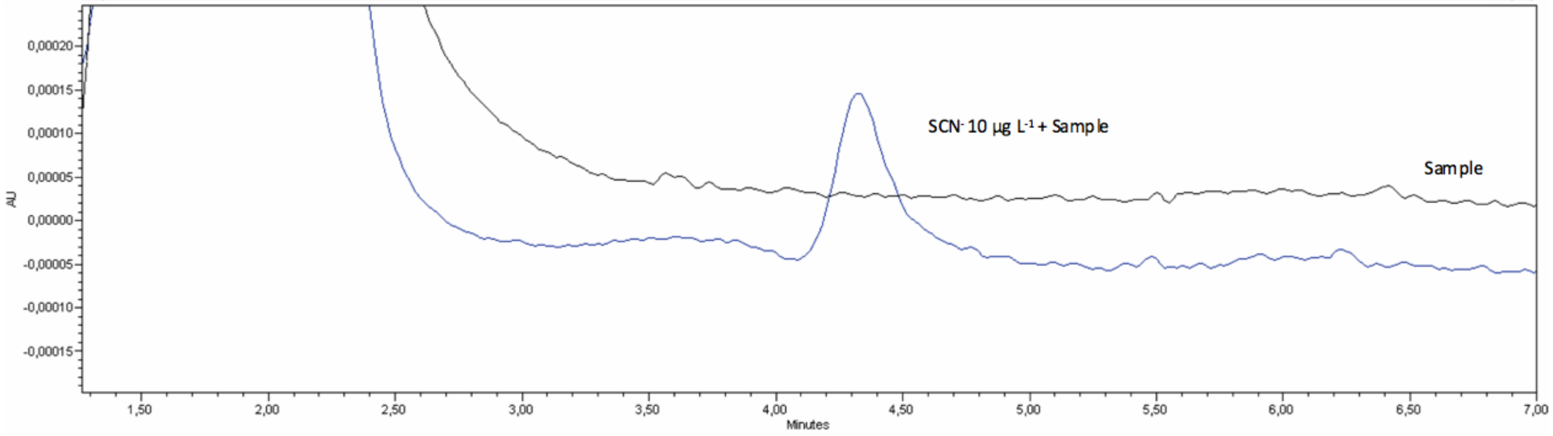
SCN- 10 µg L-1 + Sample
Sample
AU
Minutes
0,00020
0,00015
0,00010
0,00005
0,00000
-0,00005
-0,00010
-0,00015
1,50
2,00
2,50
3,00
3,50
4,00
4,50
5,00
5,50
6,00
6,50
7,00

S1bo

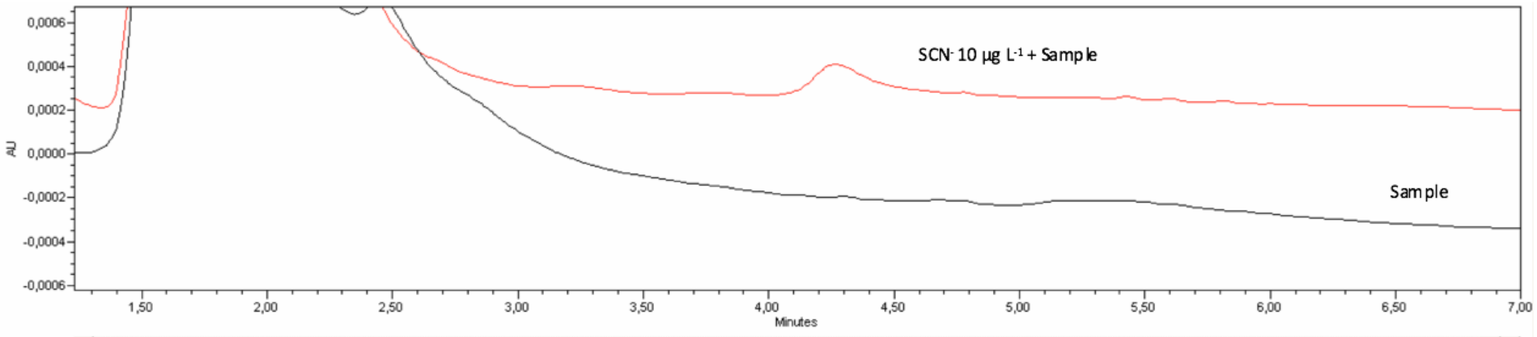
SCN- 10 µg L-1 + Sample
Sample
AU
0,0006
0,0004
0,0002
0,0000
-0,0002
-0,0004
-0,0006
1,50
2,00
2,50
3,00
3,50
4,00
4,50
5,00
5,50
6,00
6,50
7,00
Minutes

S1bp

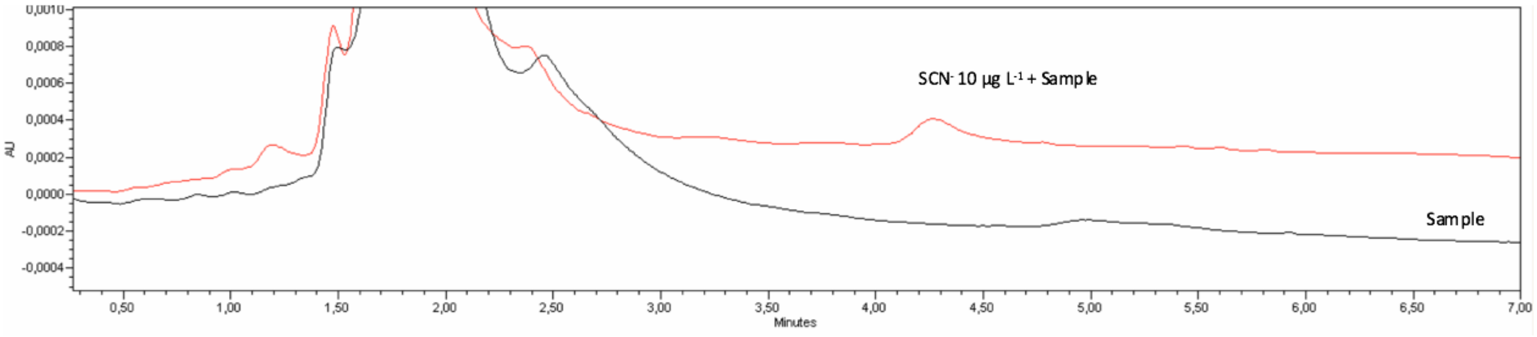
SCN- 10 µg L-1 + Sample
Sample
AU
0,0010
0,0008
0,0006
0,0004
0,0002
0,0000
-0,0002
-0,0004
0,50
1,00
1,50
2,00
2,50
3,00
3,50
4,00
4,50
5,00
5,50
6,00
6,50
7,00
Minutes

S1bq

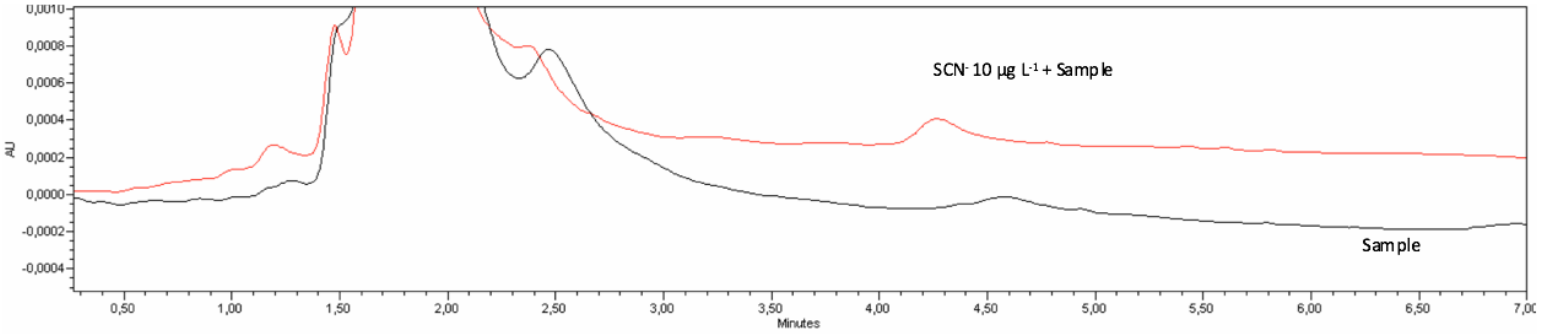

SCN- 10 µg L-1 + Sample
Sample
AU
Minutes

S1br

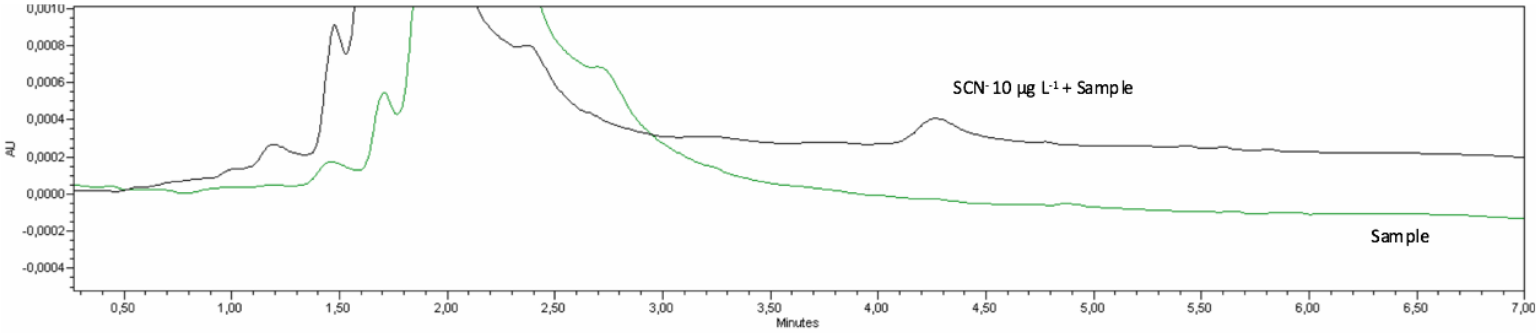

SCN- 10 µg L-1 + Sample
Sample
AU
Minutes

S1bs

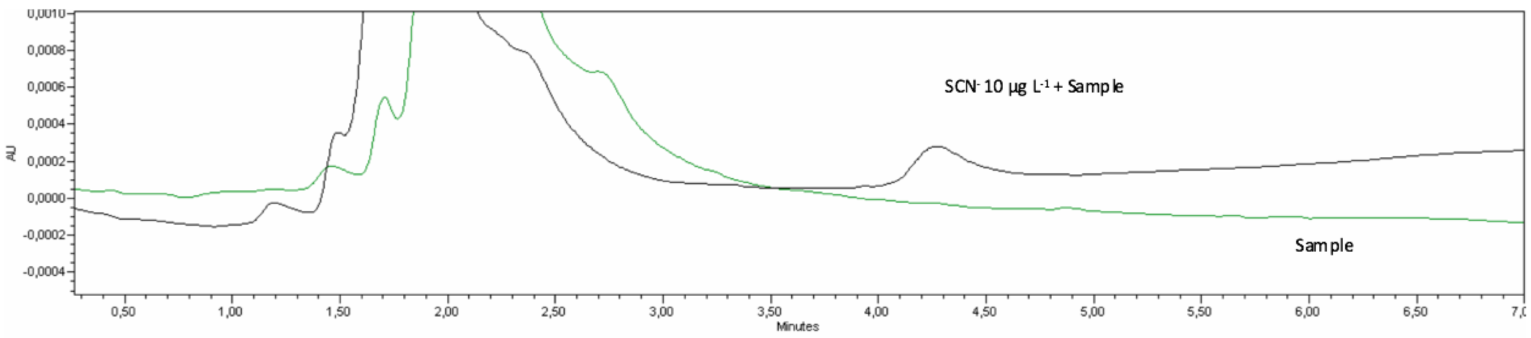
SCN- 10 µg L-1 + Sample
Sample
AU
Minutes

S1bt

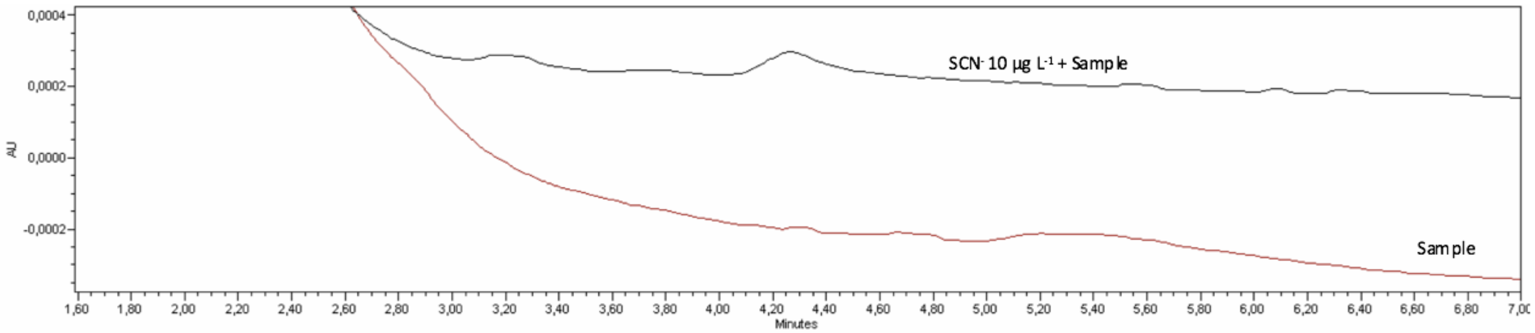
SCN- 10 µg L-1 + Sample
Sample
AU
Minutes

S1bu

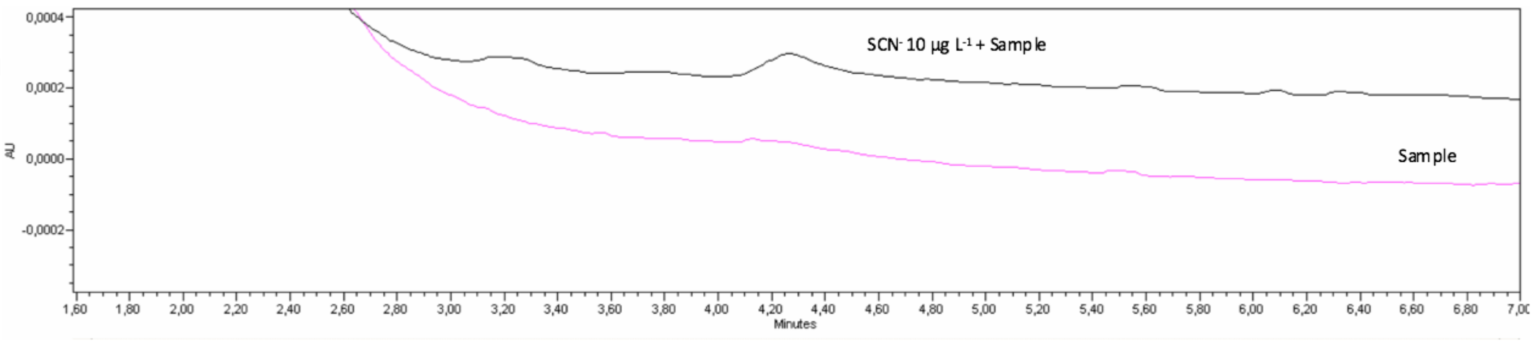
SCN⁻ 10 µg L⁻¹ + Sample
Sample
AU
Minutes

S1bv

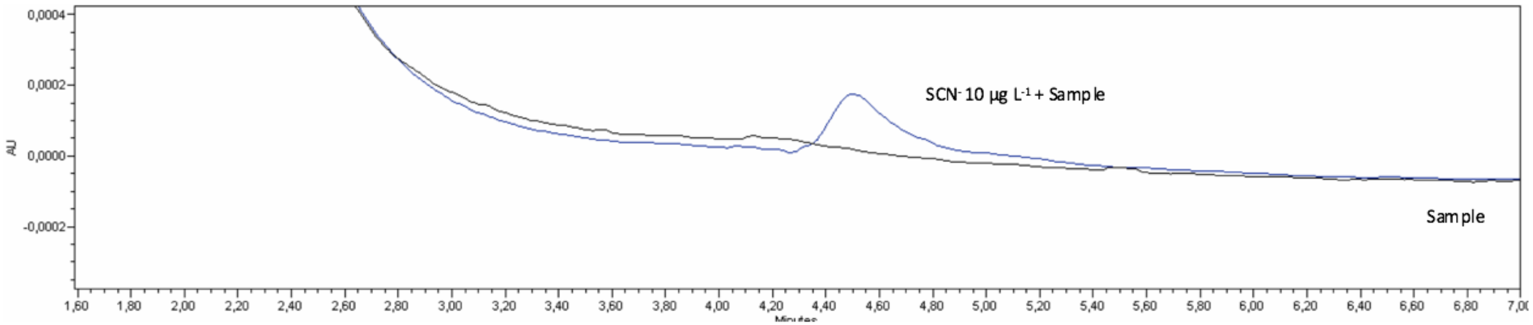
SCN⁻ 10 µg L⁻¹ + Sample
Sample
AU

Supplementary Figure S2 – Chromatograms performed using HPLC to screen for the presence of SCN^- in seawater used to depurate blue damsels *Chrysiptera cyanea* (family Pomacentridae) of similar size during 24 h. A) Chromatograms representing a sample displaying potential levels of SCN^- (sample), with identification being confirmed through the spiking of the sample with 40 µg L^{-1} of SCN^- (SCN^- 40 µg L^{-1}) and detecting a proportional increase in the peak of SCN^- present in the sampled spiked with 40 µg L^{-1} of SCN^- (Sample + SCN^- 40 µg L^{-1}); B) Chromatograms representing a sample displaying no detectable level of SCN^- (sample), with the absence of SCN^- being confirmed through the spiking of the sample with 40 µg L^{-1} of SCN^- (SCN^- 40 µg L^{-1}) and the sole detection of the peak referring to concentration of SCN^- added to the sample (Sample + SCN^- 40 µg L^{-1}); C) Chromatograms representing a sample displaying potential levels of SCN^- (sample), with the absence of SCN^- being confirmed through the spiking of the sample with 40 µg L^{-1} of SCN^- (SCN^- 40 µg L^{-1}) and the detection of two peaks referring to the concentration of SCN^- added to the sample and the ion initially detected that was not confirmed as SCN^- (Sample + SCN^- 40 µg L^{-1}). Retention times recorded for the SCN^- were always between 4.2 to 4.8 minutes, with shifts in retention times for SCN^- peaks represented in the figure being due to the stripping of PEG (Polyethyleneglycol - H(OCH2CH2)nOH) used to modify the C30 column employed for HPLC analysis.

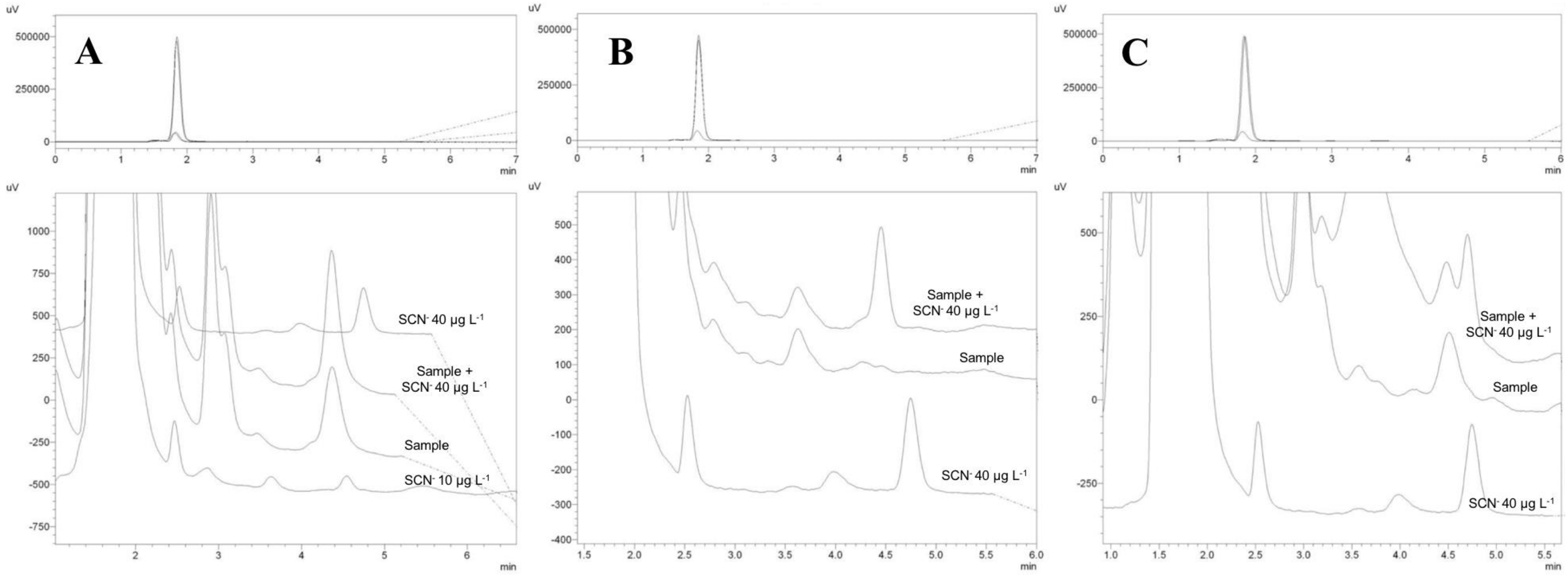

Supplementary Table S1 – HPLC chromatogram of standard solutions and all live reef fish screened for thiocyanate (SCN^-) excretion after being depurated for 24 h in synthetic seawater along with their family, scientific and common names. Scientific names of fish species and families are reported according to WoRMS[20], while common names are reported according to Michael[21].

Standart Solutions	
**Base Line**	**Retention Time**
SCN^- 40 μg L^{-1}	4,0952
SCN^- 20 μg L^{-1}	4,1013
SCN^- 10 μg L^{-1}	4,1592
SCN^- 5 μg L^{-1}	4,1491
SCN^- 20 μg L^{-1}	4,5013
SCN^- 10 μg L^{-1}	4,5503
**Average**	**4,2594**
Max value	4,5503
Min value	4,0952
Supplementary figure S1a and S1b	

Supplementary Table S1 (continued)

Chromatograms tested positive for SCN⁻ *(Detection Limit = 10 μg L⁻¹ SCN⁻)								
**Family**	**Common name**	**Species**	**Common name**	**Supplementary figure**	**Base Line**	**Retention Time**	**Base line**	**Retention Time**
**Acanthuridae**	Surgeonfishes	*Acanthurus lineatus*	Lined surgeonfish	S1c	SCN^- 20 μg L^{-1} + Sample	4,1821	Sample	3,9716
		Zebrasoma scopas	Brown tang	S1g	SCN^- 20 μg L^{-1} + Sample	4,0323	Sample	4,0321
**Chaetodontidae**	Butterflyfishes	*Chelmon rostratus*	Copperbanded butterflyfish	S1h	SCN^- 20 μg L^{-1} + Sample	4,0010	Sample	3,9563
		Chelmon rostratus		S1i	SCN^- 20 μg L^{-1} + Sample	4,2162	Sample	4,0100
		Chelmon rostratus		S1j	SCN^- 20 μg L^{-1} + Sample	4.7456	Sample	4,3798
**Monacanthidae**	Foolfish	*Acreichthys tomentosus*	Bristle-tail file-fish	S1l	SCN^- 20 μg L^{-1} + Sample	4,6770	Sample	4,6928
**Pomacentridae**	**Damselfishes**	***Chrysiptera cyanea***	**Blue damselfish**	**S2A**	**SCN^- 40 μg L^{-1} + Sample**	**4,5234**	**Sample**	**4,5765**
**Pomacanthidae**	Angelfishes	*Centropyge ferrugata*	Rusty angelfish	S1d	SCN^- 20 μg L^{-1} + Sample	4,1753	Sample	4,0924
		Centropyge ferrugata		S1e	SCN^- 20 μg L^{-1} + Sample	4,1286	Sample	4,1174
		Centropyge ferrugata		S1f	SCN^- 20 μg L^{-1} + Sample	4,1985	Sample	4,1291
**Siganidae**	Rabbitfishes	*Siganus vulpinus*	Foxface rabbitfish	S1k	SCN^- 20 μg L^{-1} + Sample	4.009	Sample	4,1110
					**Average**	**4,2372**	**Average**	**4,1881**
					Max value	4,6770	Max value	4,6928
					Min value	4,0010	Min value	3,9563

Supplementary Table S1 (continued)

Chromatograms tested negative for SCN^- * (For concentrations above 10 μg L^{-1} SCN^-)						
**Family**	**Common name**	**Species**	**Common name**	**Supplementary figure**	**Base Line**	**Retention Time**
**Acanthuridae**	Surgeonfishes	*Acanthurus lineatus*	Lined surgeonfish	S1m	SCN^- 10 μg L^{-1} + Sample	3,9964
		Acanthurus lineatus		S1n	SCN^- 10 μg L^{-1} + Sample	4,1693
		Zebrasoma scopas	Brown tang	S1s	SCN^- 10 μg L^{-1} + Sample	4,0598
		Zebrasoma scopas		S1t	SCN^- 10 μg L^{-1} + Sample	3,9321
		Zebrasoma scopas		S1u	SCN^- 10 μg L^{-1} + Sample	3,9479
		Acanthurus pyroferus	Chocolate surgeonfish	S1am	SCN^- 10 μg L^{-1} + Sample	4,5911
		Acanthurus tennenti	Doubleband surgeonfish	S1an	SCN^- 10 μg L^{-1} + Sample	4,4776
		Zebrasoma veliferum	Sailfin tang	S1ao	SCN^- 10 μg L^{-1} + Sample	4,4312
**Apogonidae**	Cardinalfishes	*Pterapogon kauderni*	Banggai cardinalfish	S1ap	SCN^- 10 μg L^{-1} + Sample	4,1345
		Sphaeramia nematoptera	Pajama cardinalfish	S1aq	SCN^- 10 μg L^{-1} + Sample	4,1444
**Balistidae**	Triggerfishes	*Odonus niger*	Niger triggerfish	S1ar	SCN^- 10 μg L^{-1} + Sample	4,1255
**Blenniidae**	Blennies	*Ecsenius bicolor*	Bicolor blenny	S1as	SCN^- 10 μg L^{-1} + Sample	4,1677
**Callionymidae**	Dragonets	*Synchiropus splendidus*	Green mandarinfish	S1at	SCN^- 10 μg L^{-1} + Sample	4,2987
**Chaetodontidae**	Butterflyfishes	*Chelmon rostratus*	Copperbanded butterflyfish	S1w	SCN^- 10 μg L^{-1} + Sample	3,9956
		Chelmon rostratus		S1y	SCN^- 10 μg L^{-1} + Sample	3,9897
		Chaetodon auriga	Threadfin butterflyfish	S1au	SCN^- 10 μg L^{-1} + Sample	4,0202
		Heniochus.sp	-	S1av	SCN^- 10 μg L^{-1} + Sample	4,3124
**Labridae**	Wrasses	*Labroides dimidiatus*	Bluestreak cleaner wrasse	S1aw	SCN^- 10 μg L^{-1} + Sample	4,5211
**Lutjanidae**	Snappers	*Lutjanus sebae*	Emperor snapper	S1ax	SCN^- 10 μg L^{-1} + Sample	4,3211
**Microdesmidae**	Dartfishes	*Nemateleotris magnifica*	Fire goby	S1v	SCN^- 10 μg L^{-1} + Sample	4,5984
		Nemateleotris magnifica		S1x	SCN^- 10 μg L^{-1} + Sample	4,6001
**Ostraciidae**	Cowfishes/Boxfishes	*Ostracion cubicus*	Cube boxfish	S1ay	SCN^- 10 μg L^{-1} + Sample	4,3031
		Ostracion cubicus		S1az	SCN^- 10 μg L^{-1} + Sample	4,3112
		Ostracion cubicus		S1ba	SCN^- 10 μg L^{-1} + Sample	4,3428

Supplementary Table S1 (continued)

Chromatograms tested negative for SCN^- * (For concentrations above 10 µg L^{-1} SCN^-)						
**Family**	**Common name**	**Species**	**Common name**	**Supplementary figure**	**Base Line**	**Retention Time**
**Pomacentridae**	**Damselfishes**	***Chrysiptera cyanea***	**Blue damselfish**	**S2B**	**SCN^- 40 µg L^{-1} + Sample**	**4,6234**
		Chrysiptera cyanea		**S2C**	**SCN^- 40 µg L^{-1} + Sample**	**4,6109**
		Chrysiptera cyanea		S1aa	SCN^- 10 µg L^{-1} + Sample	4,3210
		Chrysiptera cyanea		S1ab	SCN^- 10 µg L^{-1} + Sample	5,0090
		Chrysiptera cyanea		S1ac	SCN^- 10 µg L^{-1} + Sample	4,1230
		Chrysiptera cyanea		S1ad	SCN^- 10 µg L^{-1} + Sample	4,1807
		Chrysiptera cyanea		S1ae	SCN^- 10 µg L^{-1} + Sample	4,4098
		Chrysiptera cyanea		S1af	SCN^- 10 µg L^{-1} + Sample	4,5321
		Chrysiptera cyanea		S1ag	SCN^- 10 µg L^{-1} + Sample	4,4164
		Chrysiptera cyanea		S1ah	SCN^- 10 µg L^{-1} + Sample	4,5007
		Chrysiptera cyanea		S1ai	SCN^- 10 µg L^{-1} + Sample	4.5164
		Chrysiptera cyanea		S1aj	SCN^- 10 µg L^{-1} + Sample	4,5415
		Amphiprion ocellaris	Ocellaris anemonefish	S1be	SCN^- 10 µg L^{-1} + Sample	4,3969
		Amphiprion ocellaris		S1bf	SCN^- 10 µg L^{-1} + Sample	4,3211
		Chromis nitida	Barrier reef chromis	S1bg	SCN^- 10 µg L^{-1} + Sample	4,3121
		Chromis nitida		S1bh	SCN^- 10 µg L^{-1} + Sample	4,2987
		Chromis viridis		S1bi	SCN^- 10 µg L^{-1} + Sample	4,3228
		Chromis viridis		S1bj	SCN^- 10 µg L^{-1} + Sample	4,3239
		Chrysiptera parasema	Yellowtail blue damselfish	S1bk	SCN^- 10 µg L^{-1} + Sample	4,3183
		Chrysiptera parasema		S1bl	SCN^- 10 µg L^{-1} + Sample	4,2976
		Chrysiptera parasema		S1bm	SCN^- 10 µg L^{-1} + Sample	4,3928
		Chrysiptera parasema		S1bn	SCN^- 10 µg L^{-1} + Sample	4,3865
		Dascyllus sp.	-	S1bo	SCN^- 10 µg L^{-1} + Sample	4,2701

Supplementary Table S1 (continued)

Chromatograms tested negative for SCN^- * (For concentrations above 10 µg L^{-1} SCN^-)						
**Family**	**Common name**	**Species**	**Common name**	**Supplementary figure**	**Base Line**	**Retention Time**
**Pomacanthidae**	Angelfishes	*Pomacentrus alleni*	Allen's damselfish	S1o	SCN^- 10 µg L^{-1} + Sample	4,2274
		Pomacentrus alleni		S1p	SCN^- 10 µg L^{-1} + Sample	4,2297
		Centropyge acanthops	African flameback angelfish	S1q	SCN^- 10 µg L^{-1} + Sample	4,1345
		Pygoplites diacanthus	Regal angelfishes	S1r	SCN^- 10 µg L^{-1} + Sample	4,1445
		Centropyge bispinosa	Coral beauty	S1z	SCN^- 10 µg L^{-1} + Sample	4,6817
		Centropyge bicolor	Bicolor angelfish	S1bb	SCN^- 10 µg L^{-1} + Sample	4,2867
		Centropyge bicolor		S1bc	SCN^- 10 µg L^{-1} + Sample	4,2952
		Centropyge bicolor		S1bd	SCN^- 10 µg L^{-1} + Sample	4,3077
**Scaridae**	Parrotfishes	*Scarus quoyi*	Quoy's parrotfish	S1bp	SCN^- 10 µg L^{-1} + Sample	4,2551
**Serranidae**	Anthias	*Pseudanthias squamipinnis*	Lyretail anthias	S1bq	SCN^- 10 µg L^{-1} + Sample	4,3210
		Pseudanthias squamipinnis		S1br	SCN^- 10 µg L^{-1} + Sample	4,3190
		Pseudanthias squamipinnis		S1bs	SCN^- 10 µg L^{-1} + Sample	4,2700
**Siganidae**	Rabbitfishes	*Siganus vulpinus*	Foxface rabbitfish	S1bt	SCN^- 10 µg L^{-1} + Sample	4,3525
		Siganus vulpinus		S1bu	SCN^- 10 µg L^{-1} + Sample	4,2123
**Syngnathidae**	Pipefishes	*Corythoichthys intestinalis*	Messmate pipefish	S1ak	SCN^- 10 µg L^{-1} + Sample	4,2409
**Tetraodontidae**	Pufferfishes	*Arothron hispidus*	Whitespotted	S1al	SCN^- 10 µg L^{-1} + Sample	4,1767
		Canthigaster valentini	Saddled toby	S1bv	SCN^- 10 µg L^{-1} + Sample	4,5102
					**Average (from all negatives)**	**4,3122**
					Max value	5,0090
					Min value	3,9321

Supplementary Table S2 – Synthetic seawater volume employed to depurate selected fish according to their total length (measured from the tip of the snout to the tip of the longer lobe of the caudal fin).

**Fish total length (TL) (mm)**	**Seawater volume (ml)**
TL < 1,5	500
1,5 < TL > 3	1000
3 < TL > 5	1500
5 < TL > 10	2000
10 < TL > 20	5000
